# Prediction of acute kidney injury in patients with acute pesticide poisoning using the PKIP score

**DOI:** 10.1038/s41598-026-41334-4

**Published:** 2026-03-26

**Authors:** Younghee Kim, Se-Jin Ahn, Nam-Jun Cho, Inyong Jeong, Bomi Choi, Dong-Jin Lee, Samuel Park, Eun Young Lee, Hwamin Lee, Hyo-Wook Gil

**Affiliations:** 1 Department of Internal Medicine, Soonchunhyang University Cheonan Hospital, 31 Suncheonhyang 6-Gil, Dongnam-Gu, Cheonan, 31151 Republic of Korea; 2 Department of Biomedical Informatics, Korea University College of Medicine, Seoul, Republic of Korea

**Keywords:** Pesticides, Poisoning, Acute kidney injury, Mortality, Decision support techniques, Kidney diseases, Toxicology, Mathematics and computing

## Abstract

Acute pesticide poisoning frequently leads to acute kidney injury (AKI), which is strongly associated with increased mortality. However, predictive research in this area remains limited, and criteria for AKI detection in patients with pesticide poisoning are not well-defined. This study aimed to evaluate the Kidney Disease: Improving Global Outcomes (KDIGO) criteria and develop a model for early AKI prediction in patients with pesticide poisoning. This retrospective study analyzed 877 patients presenting with acute pesticide poisoning between 2015 and 2020. AKI was defined using KDIGO criteria, considering serum creatinine, urine output, and renal replacement therapy initiation. Six machine learning models with four feature selection methods were compared using fivefold cross-validation, stratified by pesticide category. The final model, Prediction of acute Kidney Injury in Pesticide intoxication (PKIP), was established. KDIGO-defined AKI was significantly associated with mortality, with AKI patients showing a 16.6% mortality compared to 4.7% in non-AKI patients. The PKIP model, incorporating 14 features selected via the Least Absolute Shrinkage and Selection Operator, demonstrated fair discrimination [AUROC 0.720 (95% CI: 0.692–0.747), AUPRC 0.513 (95% CI: 0.464–0.563)]. Furthermore, the model showed prognostic utility for mortality prediction [AUROC 0.839 (95% CI: 0.767–0.910), AUPRC 0.421 (95% CI: 0.246–0.595)]. At the predefined cutoff value of 0.420, the model achieved a sensitivity of 39.0% and a specificity of 89.7%. Risk stratification based on PKIP probabilities showed significant differences in outcomes between groups. The high-risk group demonstrated significantly higher risks of AKI occurrence, progression to higher AKI stages, and mortality compared to the low-risk group. PKIP exhibited superior risk stratification for both AKI and mortality prediction compared to the APACHE II score. This study validates the use of KDIGO criteria for AKI detection in pesticide poisoning and introduces the PKIP model as a tool demonstrating moderate discrimination for early AKI prediction and risk stratification. The web-based PKIP tool can serve as a practical instrument for clinical decision-making for patients with pesticide poisoning. Future research should focus on external validation of the PKIP model and assessment of its impact on patient outcomes in diverse clinical settings.

*Trial registration*: Retrospectively registered.

**Supplementary Information:**

The online version contains supplementary material available at 10.1038/s41598-026-41334-4.

## Introduction

Acute pesticide poisoning is associated with a high risk of mortality; severe cases often lead to multi-organ damage affecting the lungs, heart, and kidneys^1^. Among these, acute kidney injury (AKI) is a critical determinant of poor prognosis in hospitalized patients. Although several studies have shown that AKI is significantly associated with an increased risk of mortality in patients with acute poisoning, research focused on predicting or investigating AKI in this group is limited^2^.

In cases of pesticide poisoning, AKI can affect prognosis in various ways^3^. The kidneys play a critical role in toxin elimination, and their functional status considerably influences the toxicity of substances^4,5^. Therefore, accurately predicting AKI and identifying predisposing factors are crucial for establishing therapeutic strategies to preserve renal function in patients with acute pesticide poisoning.

Although AKI is a crucial factor in this context, a unified definition for AKI specific to pesticide poisoning is still not established. The definition of acute renal failure has evolved over the past decade through the development of classification systems like Risk, Injury, Failure, Loss of kidney function, and End-stage kidney disease (RIFLE), introduced by the Acute Dialysis Quality Initiative (ADQI), the Acute Kidney Injury Network (AKIN) classification, and the Kidney Disease: Improving Global Outcomes (KDIGO) guidelines^6^. The KDIGO criteria are widely accepted for defining AKI, and recent research has investigated their applicability across various clinical settings and organ systems^7–9^. However, evidence regarding the suitability of these definitions for patients with acute poisoning remains limited^10^. For example, the Poisoning Severity Score, which defines acute renal failure when serum creatinine (sCr) levels exceed 200 µmol/L, may no longer align with the latest understanding of AKI^11^.

It is crucial to evaluate the suitability of utilizing KDIGO-based AKI definitions in patients with acute pesticide poisoning and to assess their prognostic relevance. Developing an early prediction model for AKI in this population could support earlier risk stratification and potentially inform clinical decision-making aimed at preserving renal function. Therefore, this study has two primary objectives: (1) to evaluate the applicability of KDIGO criteria for defining AKI in patients with acute pesticide poisoning and assess its prognostic significance for mortality, and (2) to develop and validate an early prediction model (PKIP score) that can identify patients at high risk of developing AKI within the first 2 h of presentation, thereby supporting clinical decision-making, including appropriate monitoring strategies and prioritization of care in real-world settings.

## Materials and methods

### Study population

This retrospective cohort study was conducted at the Pesticide Poisoning Research Center of Soonchunhyang University Cheonan Hospital from January 2015 to December 2020. Adult patients (≥ 19 years) who ingested pesticides were included. Patients with paraquat poisoning were excluded. Paraquat is banned in most countries due to its high toxicity and fatality primarily caused by pulmonary fibrosis^12,13^. Although paraquat-induced AKI is common, its inclusion could artificially inflate model performance without clear clinical relevance, given the high mortality and lack of routine usage^14^. Therefore, only non-paraquat pesticide poisoning cases were included. Patients with insufficient data to define baseline sCr levels or with baseline sCr levels exceeding 2.5 mg/dL were excluded. Patients who developed AKI or died (or were discharged) within 2 h of presentation were also excluded.

Comprehensive data were extracted from the electronic health records (EHR) using standardized data collection forms for the final confirmed patient cohort. The collected variables encompassed a wide range of information pertinent to acute pesticide poisoning and the prediction of AKI. The collected data included the following categories:

- Demographic and Vital Signs Demographic information such as age, sex, and Body Mass Index (BMI), along with vital sign data including systolic/diastolic blood pressure, pulse rate, respiratory rate, and Body Temperature (BT).
- Comorbidities and History Information on patients’ underlying conditions such as diabetes, hypertension, pulmonary disease, cardiovascular disease, Chronic Kidney Disease (CKD), and neuropsychiatric disorders, as well as alcohol history and presence of vomiting.
- Pesticide Poisoning-Specific Information Variables that were difficult to confirm directly from the EHR—such as pesticide type, pesticide category, amount of pesticide ingestion, exposure type, and time of poisoning—were separately compiled and extracted from standardized data collection forms completed at initial presentation or from clinical notes.
- Laboratory and Biochemical Data Extensive laboratory data derived from blood tests, arterial blood gas analysis, and electrolyte tests were included. This covered variables such as White Blood Cell (WBC) count, Hemoglobin (Hb), sCr, glucose, liver enzymes (aspartate aminotransferase, alanine aminotransferase, alkaline phosphatase [ALP]), Bicarbonate, Phosphate, Anion Gap (AG), lactate, pH, pCO_2_, pO_2_ from arterial blood gas analysis, and Urinalysis results included urine red blood cell (RBC) counts.
- Clinical Assessment and Treatment Information Information on clinical assessments like the Glasgow Coma Scale (GCS) scores, presence of hypoxemia, hypercapnia, and data on initial treatments such as gastric lavage, charcoal administration, and extracorporeal treatment (e.g., dialysis).

### Definition and staging of acute kidney injury

AKI was defined according to the KDIGO criteria^15^. AKI was classified into three stages based on sCr levels, urine output (UO), and dialysis information. All criteria for defining AKI were based on measurements taken after hospital presentation. Baseline sCr was dynamically defined as the lowest value measured within the preceding 48 h (for absolute sCr increase $\ge$ 0.3 mg/dL) or 168 h (for sCr increase $\ge$ 1.5 times baseline) during hospitalization. If a dynamic baseline sCr could not be established, the minimum sCr value measured within the first 2 h of presentation was used as a substitute. This conservative approach aligns with methodologies used in prior research^16–18^. As previously described, patients lacking baseline data or those who developed AKI, died, or were discharged within the initial 2 h of presentation were excluded.

**Fig. 1:**
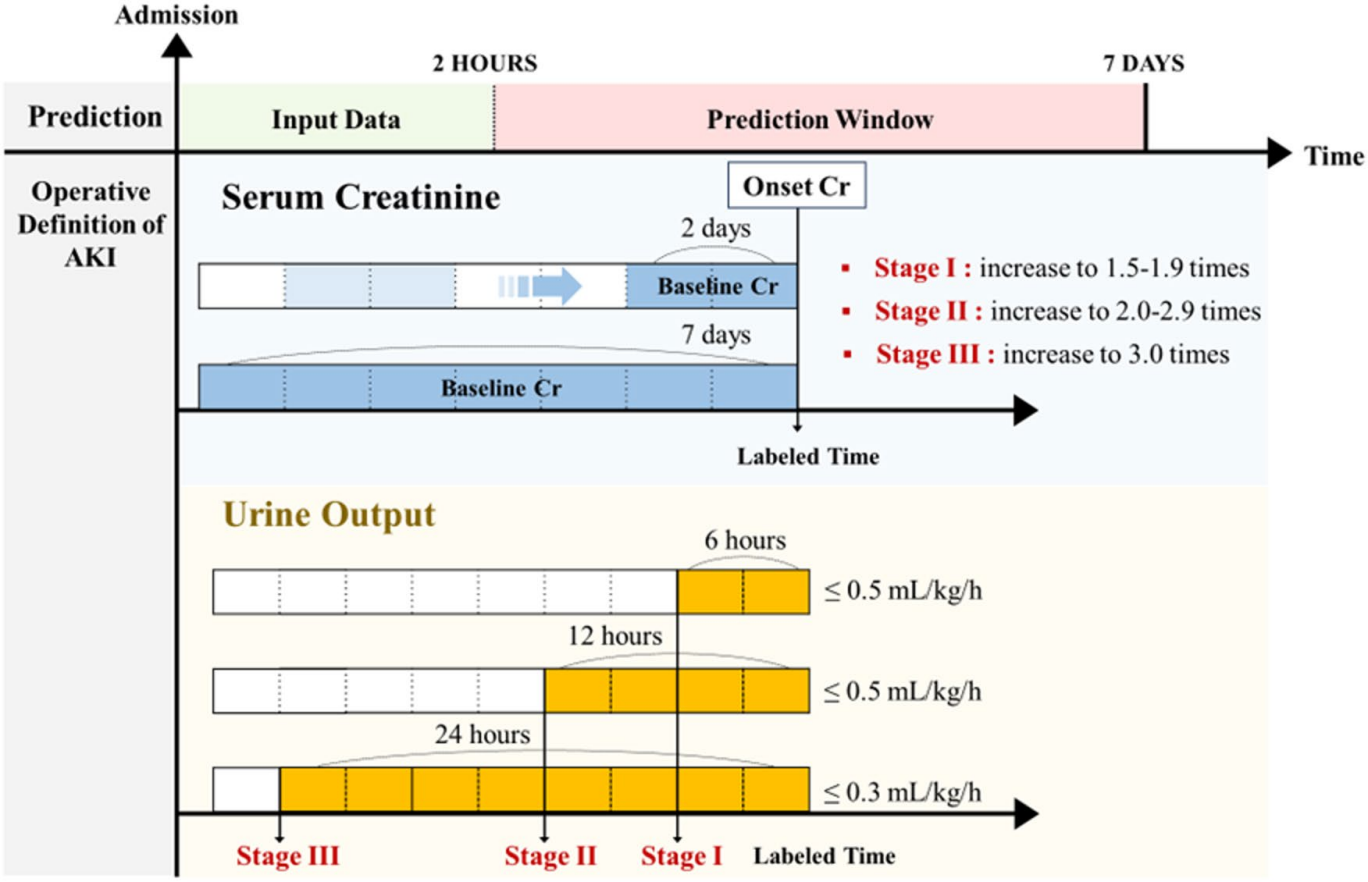
Definition of Acute Kidney Injury.

AKI staging followed standard guidelines: Stage 1 was defined as an sCr increase of 1.5–1.9 times baseline; Stage 2 as 2.0–2.9 times baseline; and Stage 3 as $\ge$ 3.0 times baseline or the initiation of maintenance dialysis. For UO, a moving window approach was applied for 6-, 12-, and 24-h intervals, normalized by patient weight. UO criteria were defined as follows: Stage 1 (< 0.5 mL/kg/h for 6 h), Stage 2 (< 0.5 mL/kg/h for 12 h), and Stage 3 (< 0.3 mL/kg/h for 24 h or anuria for 12 h). The final AKI stage was determined by the highest stage identified within 168 h across sCr, UO, or dialysis criteria. The time of AKI onset was defined as the earliest point of identification by any criterion. This approach is schematically represented in Fig. 1.

### Study design and data preprocessing

We stratified the risk of AKI occurrence within 168 h of presentation into five categories (minimal, low, moderate, high, and severe) using data collected within the first 2 h. This 2-h window was chosen because prompt results are essential for clinical decision-making and are recommended to be obtained within the first two hours of emergency department arrival^19^. AKI occurring beyond 168 h was excluded, as late-onset AKI was considered less likely to be directly attributable to pesticide intoxication and was therefore excluded to focus on early toxic effects.

Data collected within 2 h of presentation were aggregated using the maximum value for each variable. This approach aligns with previous studies demonstrating that "worst-case" values (e.g., peak vital signs indicating early toxicity severity) often provide superior predictive performance compared to single measurements^20–22^. Conversely, laboratory tests were rarely repeated within this window (approximately 2% of patients); thus, the choice of summary statistic had negligible impact on these variables.

Missing data were handled as follows: categorical variables were imputed with the mode, and numerical variables were imputed using multiple imputation by chained equations. The frequency of missing values for each predictor is detailed in Supplementary Material 1. Finally, numerical variables underwent robust scaling.

### Univariate cox regression analysis and feature selection

Univariate Cox regression analysis was performed to identify variables associated with AKI occurrence from approximately 50 candidate variables. Variables with a p-value < 0.1 in the univariate analysis were included in the initial feature set. To refine the selection of predictors and optimize the predictive model, four feature selection methods were employed^23^:

- Full variable approach: Including all potential predictive variables in the initial model to assess the contribution of each variable.
- Clinical expertise-based selection: Prioritizing variables deemed clinically important by nephrologists.
- Stepwise selection: Combining forward and backward elimination methods to iteratively add or remove variables based on statistical significance.
- Least Absolute Shrinkage and Selection Operator (LASSO) regression: Selecting the most relevant variables while shrinking coefficients and preventing overfitting through regularization

### Development and validation of classification models

Six machine learning models were developed to predict AKI: logistic regression, random forest, XGBoost, LightGBM, CatBoost (CAT), and support vector machine. Each model was trained using variables selected via the four feature selection methods. We employed fivefold cross-validation stratified by pesticide type and AKI occurrence to ensure a balanced representation of diverse pesticide categories and AKI occurrence across all folds. Hyperparameter tuning was performed using grid search to optimize model performance. The specific ranges of hyperparameters tested for each model are provided in Supplementary Material 2.

For the selected model, accuracy, precision, recall, and F1-score were evaluated across a range of thresholds, and the threshold that optimized accuracy was chosen. Calibration curves were generated for the final optimal model, and the Brier score was calculated to assess the agreement between predicted probabilities and observed outcomes.

### Statistical analysis

Statistical analyses were performed using Python 3.11.7 and R 4.0.0 (R Foundation for Statistical Computing, Vienna, Austria). Continuous variables were summarized as median (interquartile range) or mean (standard deviation) depending on their distribution, while categorical variables were presented as frequencies and percentages.

The normality of continuous variables was assessed using the Shapiro–Wilk test, and homogeneity of variance was evaluated using Levene’s test. Group comparisons were performed using Student’s t-test for normally distributed variables and the Mann–Whitney U test for non-normally distributed variables. Categorical variables were compared using the χ2 test or Fisher’s exact test, as appropriate.

Kaplan–Meier survival analysis was performed to evaluate the relationship between risk groups and clinical outcomes. For mortality analysis, the time to death was defined as the time from presentation to death for deceased patients, while survivors were censored at the time of discharge. Similarly, for AKI incidence analysis, the time from presentation to AKI occurrence was calculated for patients who developed AKI, and time to discharge was used for those who did not.

Survival curves were compared across different AKI stages and risk groups for both AKI occurrence and mortality using the log-rank test. Additionally, the Cochran-Armitage trend test was conducted to evaluate the progressive increase in risk across the stratified groups. The significance level was set at *p* < 0.05 for all statistical tests.

### Code availability

The custom code used for model development and analysis in this study is publicly available at GitHub (https://github.com/5454dls/pkip-score) and archived in Zenodo (10.5281/zenodo.18611110). A web-based tool developed based on the final prediction model is described in Additional file 2. Access to the web interface is available upon reasonable request to the corresponding author.

## Results

### Characteristics of the study population

**Fig. 2:**
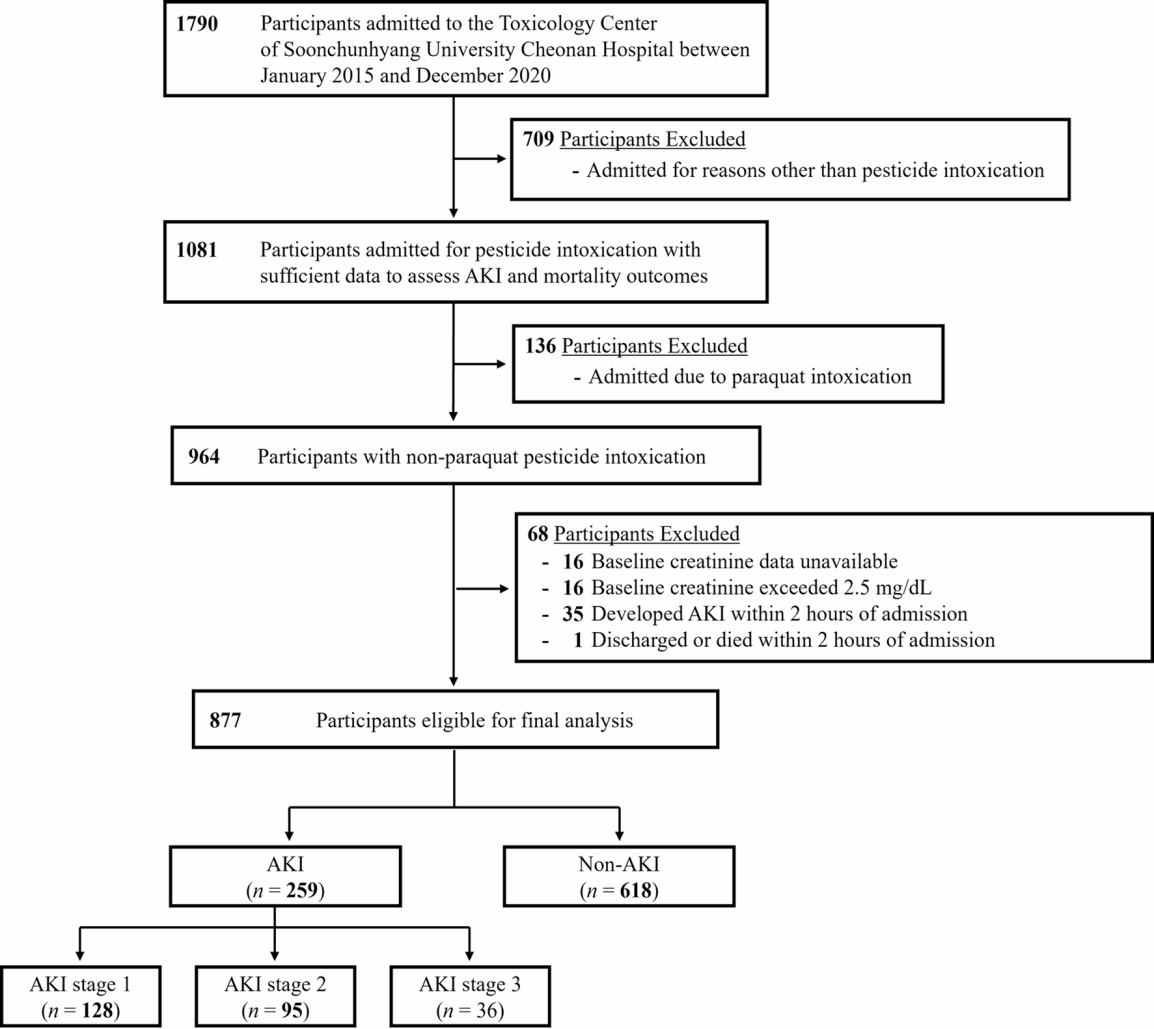
Flowchart of Patient Selection and Cohort Construction for Acute Pesticide Poisoning Study.

Figure 2 illustrates the process of cohort formation. In this retrospective cohort study of 877 patients with acute pesticide poisoning, 259 (29.5%) developed AKI within 7 days of presentation. The timeline of AKI occurrences is presented in Supplementary Material 3. Table 1 outlines the baseline characteristics of the study participants, stratified by AKI occurrence.

**Table 1:** Baseline characteristics of study participants.

Features	All Patients	AKI	Non-AKI	*P*-value
	(*n* = 877)	(*n* = 259)	*(n* = 618)	
Age, years	62.0 (51.0, 76.0)	68.0 (54.0, 79.5)	60.0 (49.0, 74.0)	0.000***
Sex, male (%)	541 (61.7)	168 (64.9)	373 (60.4)	0.239
Body Mass Index, kg/m^2^	22.5 (20.4, 24.7)	22.9 (20.8, 25.5)	22.2 (20.3, 24.4)	0.007**
Alcohol history, yes (%)	413 (47.9)	114 (44.0)	299 (49.5)	0.160
Diabetes, present (%)	165 (18.9)	49 (18.9)	116 (18.9)	1.000
Hypertension, present (%)	332 (38.0)	102 (39.4)	230 (37.4)	0.634
Lung disease, present (%)	81 (9.3)	28 (10.8)	53 (8.6)	0.372
Cardiovascular disease, present (%)	55 (6.3)	16 (6.2)	39 (6.3)	1.000
Chronic kidney disease, present (%)	8 (0.9)	6 (2.3)	2 (0.3)	0.015*
Neuropsychiatry disease, present (%)	180 (20.6)	58 (22.4)	122 (19.8)	0.446
Hypoxemia, present (%)	84 (9.6)	34 (13.2)	50 (8.1)	0.029*
Hypercapnia, present (%)	263 (30.1)	76 (29.5)	187 (30.4)	0.843
GCS score	14.8 (13.0, 15.0)	14.0 (8.6, 15.0)	15.0 (14.0, 15.0)	0.000***
APACHE II score	8.0 (5.0, 12.1)	11.0 (7.0, 17.0)	7.0 (5.0, 11.0)	0.000***
*.*				
Organophosphate, Carbamate or Glufosinate	333 (38.0)	128 (49.4)	205 (33.2)	< 0.001**
Other pesticides^a^	544 (62.0)	131 (50.6)	413 (66.8)	
*Amount of ingestion*				
Under 100 cc	340 (38.8)	78 (30.1)	262 (42.4)	0.001**
100–300 cc	318 (36.3)	107 (41.3)	211 (34.1)	
Over 300 cc	123 (14.0)	42 (16.2)	81 (13.1)	
Unknown	96 (10.9)	32 (12.4)	64 (10.4)	
Systolic blood pressure, mmHg	140.0 (120.0, 150.0)	140.0 (120.0, 160.0)	140.0 (120.0, 150.0)	0.782
Pulse rate, beats/min	90.0 (80.0, 101.0)	92.0 (82.0, 106.0)	90.0 (80.0, 100.0)	0.020*
Respiratory rate, breaths/min	20.0 (19.0, 22.0)	20.0 (19.0, 22.0)	20.0 (19.0, 21.0)	0.915
Body temperature, ˚C	36.6 (36.2, 37.0)	36.5 (36.1, 36.9)	36.7 (36.3, 37.0)	0.032*
Hemoglobin, g/dL	14.0 (12.9, 15.3)	14.1 (13.0, 15.3)	14.0 (12.8, 15.3)	0.085
White Blood Cell count, 10^3^/μL	10.1 (7.5, 14.4)	12.3 (8.7, 16.8)	9.6 (7.2, 13.4)	0.000***
Platelet count, 10^3^/μL	238.0 (201.0, 289.0)	234.0 (192.0, 280.0)	240.0 (203.0, 292.0)	0.065
Albumin mg/dL	4.3 (3.9, 4.6)	4.3 (3.9, 4.6)	4.3 (4.0, 4.6)	0.218
Glucose, mg/dL	129.0 (107.0, 161.0)	139.0 (114.0, 179.5)	126.0 (105.0, 155.8)	0.001**
Creatinine mg/dL	0.8 (0.7, 1.0)	0.9 (0.7, 1.2)	0.8 (0.7, 1.0)	0.000***
Uric Acid, mg/dL	5.2 (4.2, 6.4)	5.4 (4.4, 6.7)	5.1 (4.0, 6.4)	0.014*
Phosphate, mg/dL	3.4 (2.8, 4.1)	3.6 (2.9, 4.3)	3.3 (2.8, 4.0)	0.004**
Sodium, mmol/L	142.0 (140.0, 144.0)	142.0 (140.0, 144.0)	142.0 (139.0, 144.0)	0.149
Potassium, mmol/L	4.0 (3.7, 4.3)	3.9 (3.6, 4.4)	4.0 (3.7, 4.3)	0.492
Calcium, mg/dL	9.0 (8.5, 9.4)	8.9 (8.4, 9.4)	9.0 (8.5, 9.4)	0.252
C-Reactive Protein, mg/L	1.2 (0.5, 3.5)	1.3 (0.6, 3.8)	1.1 (0.5, 3.3)	0.930
Arterial pH	7.4 (7.3, 7.4)	7.4 (7.3, 7.4)	7.4 (7.3, 7.4)	0.000***
Partial pressure of carbon dioxide, mmHg	38.0 (34.0, 43.0)	38.0 (34.0, 43.0)	38.0 (34.0, 42.0)	0.212
Partial pressure of oxygen, mmHg	87.0 (75.0, 102.0)	88.7 (72.2, 107.0)	87.0 (76.0, 100.0)	0.082
Bicarbonate , mmol/L	22.5 (19.4, 24.8)	21.1 (18.2, 23.9)	22.9 (20.2, 25.4)	0.000***
Anion gap, mmol/L	16.0 (13.0, 19.5)	16.9 (13.7, 22.1)	15.7 (12.9, 18.7)	0.000***
Alkaline phosphatase, U/L	72.0 (58.0, 88.0)	75.0 (60.0, 93.0)	71.0 (57.0, 86.0)	0.009**
Aspartate transaminase, U/L	27.0 (21.0, 39.0)	30.0 (24.0, 46.0)	26.0 (21.0, 36.0)	0.030*
Blood urea nitrogen, mg/dL	14.6 (11.2, 18.5)	15.4 (11.9, 19.5)	14.1 (10.6, 18.2)	0.000***
Urine specific gravity	1.0 (1.0, 1.0)	1.0 (1.0, 1.0)	1.0 (1.0, 1.0)	0.323
Triglyceride, mg/dL	135.0 (88.0, 231.0)	139.0 (96.0, 261.0)	131.0 (86.0, 217.5)	0.196
Total bilirubin, mg/dL	0.5 (0.3, 0.7)	0.5 (0.3, 0.8)	0.5 (0.3, 0.7)	0.038*
Total cholesterol, mg/dL	171.0 (145.0, 201.0)	170.0 (145.0, 205.0)	171.0 (145.0, 200.0)	0.939
Total protein, g/dL	7.0 (6.5, 7.4)	7.0 (6.5, 7.4)	6.9 (6.5, 7.4)	0.980
Lactate, mmol/L	2.6 (1.5, 4.4)	3.2 (1.8, 5.4)	2.4 (1.5, 4.1)	0.000***
Activated Partial Thromboplastin Time, seconds	27.7 (24.8, 30.7)	27.6 (24.7, 30.2)	27.8 (24.9, 30.8)	0.988
Prothrombin Time—International Normalized Ratio	1.0 (1.0, 1.1)	1.0 (1.0, 1.1)	1.0 (1.0, 1.1)	0.584
*Urine RBC, cells*				
< 1	362 (41.3)	88 (34.0)	274 (44.3)	0.006**
≥ 1	515 (58.7)	171 (66.0)	344 (55.7)	
*Urine protein*				
Negative	464 (52.9)	115 (44.4)	349 (56.5)	0.001**
Trace	341 (38.9)	116 (44.8)	225 (36.4)	
≥ 1	72 (8.2)	28 (10.8)	44 (7.1)	
*Acute kidney injury*				
Non—AKI	618 (70.5)	–	618 (100.0)	–
AKI stage 1	128 (14.6)	128 (49.4)	–	
AKI stage 2	95 (10.8)	95 (36.7)	–	
AKI stage 3	36 (4.1)	36 (13.9)	–	
Death ^b^, yes (%)	72 (8.2)	43 (16.6)	29 (4.7)	0.000***

Data are presented as medians with interquartile ranges for continuous variables and as numbers with percentages for categorical variables. GCS, Glasgow Coma Scale; APACHE, Acute Physiology and Chronic Health Evaluation; RBC, Red Blood Cell; AKI, Acute Kidney Injury.

^a^Other pesticides include acetanilide, acetylaniline, alryoxylcarboxide, amide, anilin, arsenic, phenopropionate, benzohydrazide, benzoate, chlorfenapyr, chloroacetamide, chloronicotinyl, diamide, diazine, dinitroaniline, endosulfan, fungicide, insect growth regulator, lambda cyhalothrin, neonicotinoid, niacin, oxadiazole, phenoxy, pyrol, sulfonylurea, sulfoximine, sulfuryl fluoride, tetramic acid, tetrazolium oxide, and unknown pesticides.

^b^Death refers to all-cause mortality.

*indicates *p*-value < 0.05.

**indicates *p*-value < 0.01.

***indicates *p*-value < 0.001.

Patients who developed AKI were significantly older (median age 68.0 vs. 60.0 years, *p* < 0.001) and had a higher BMI (median 22.9 vs. 22.2 kg/m^2^, *p* = 0.007) compared to those without AKI. The prevalence of CKD was also higher in the AKI group (2.3% vs. 0.3%, *p* = 0.015).

Severity indicators showed significant differences between the groups. Patients with AKI had lower GCS scores (median 14.0 vs 15.0,* p* < 0.001). The mortality was substantially higher in the AKI group (16.6% vs 4.7%, *p* < 0.001).

Regarding pesticide types, organophosphates, carbamates, or glufosinate were more frequently associated with AKI development (49.4% vs 33.2%, *p* < 0.001). Patients who ingested larger amounts of pesticides (100–300 cc) showed a trend toward higher AKI incidence (41.3% vs 34.1%, *p* = 0.053).

Laboratory findings revealed several significant differences. Patients who developed AKI had higher WBC counts (median 12.3 vs 9.6 × 10^3^/μL, *p* < 0.001), glucose levels (median 139.0 vs 126.0 mg/dL, p = 0.001), and sCr (median 0.9 vs 0.8 mg/dL, *p* < 0.001). They also showed lower bicarbonate levels (median 21.1 vs 22.9 mmol/L, *p* < 0.001) and higher AG (median 16.9 vs 15.7, *p* < 0.001). Notably, lactate levels were significantly higher in the AKI group (median 3.2 vs 2.4 mmol/L, *p* < 0.001). Urinalysis results indicated that patients who developed AKI were more likely to have abnormal findings. Patients who developed AKI had a higher percentage of elevated urine RBC counts. (66.0% vs 55.7%, *p* = 0.006), and a lower frequency of negative urine protein results.

### Survival analysis

**Fig. 3:**
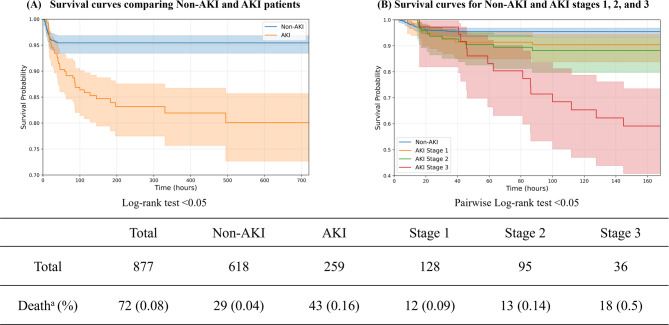
Kaplan–Meier survival curves for mortality based on AKI status and stages.

Figure 3 presents the survival analysis results for mortality based on AKI occurrence and AKI stages. The analysis revealed that patients who developed AKI had significantly higher mortality compared to those without AKI (*p* < 0.005).

Furthermore, a clear trend of increasing mortality was observed as the AKI stage progressed. Pairwise log-rank tests between all AKI stages (Stage 0 vs. 1, 0 vs. 2, 0 vs. 3, 1 vs. 2, 1 vs. 3, and 2 vs. 3) consistently yielded p values < 0.005. These results indicate statistically significant differences in survival rates across all AKI stages, suggesting that higher stages of AKI are associated with poorer prognosis.

### Univariate analysis results

The results of the univariate Cox regression analysis are presented in Supplementary Material 4. Several variables showed significant associations with AKI development, including demographic factors such as age (hazard ratios [HR] 1.02, 95% confidence interval [CI] 1.01–1.02, *p* < 0.001) and BMI (HR 1.05, 95% CI 1.01–1.09, *p* = 0.006), clinical parameters like GCS scores (HR 0.89, 95% CI 0.86–0.92, *p* < 0.001), and laboratory findings including WBC count (HR 1.04, 95% CI 1.02–1.06, *p* < 0.001), sCr (HR 2.93, 95% CI 2.03–4.22, *p* < 0.001), and arterial pH (HR 0.97 per 0.01 increase, 95% CI 0.95–0.98, p < 0.001).

Notably, the type of pesticide ingested was significantly associated with AKI risk, with organophosphates, carbamates, or glufosinate showing a higher risk compared to other pesticides (HR 1.68, 95% CI 1.30–2.17, *p* < 0.001). Additional univariate Cox regression analyses stratified by pesticide groups are presented in Supplementary Material 5.

### Feature selection and model comparison

The variables selected by each method and the hyperparameters for six machine learning models are presented in Supplementary Material 6. A comprehensive comparison of model performance across different variable selection methods is provided in Supplementary Material 7. This table presents the Area Under the Receiver Operating Characteristic curve (AUROC) and Area Under the Precision-Recall Curve (AUPRC) values with 95% CI for each combination of feature selection method and machine learning model. Based on these results, we selected the CAT algorithm with LASSO-selected features as PKIP model, as it achieved the highest AUROC of 0.720 (95% CI: 0.693—0.747) and AUPRC of 0.513 (95% CI: 0.464—0.563).

The LASSO method selected binary features (hypoxemia, CKD), numerical features (age, BMI, BT, GCS scores, bicarbonate, phosphate, WBC count, Hb, ALP), and categorical features (pesticide category, urine RBC).

### Final model performance

**Fig. 4:**
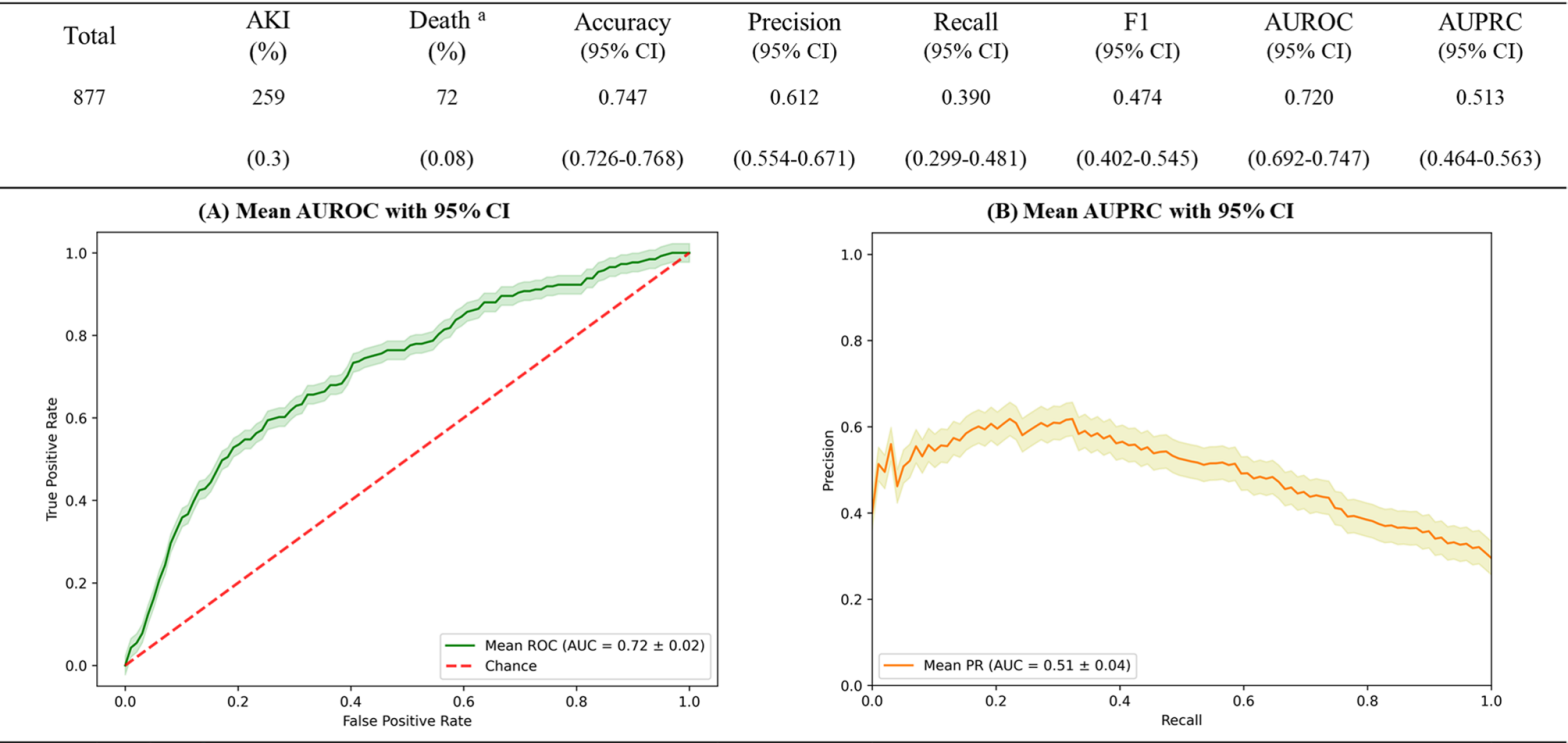
Performance metrics of the Prediction of acute Kidney Injury in Pesticide intoxication (PKIP) model.

The PKIP model’s performance was evaluated across different thresholds, with optimal accuracy achieved at a threshold of 0.420. The performance metrics are presented in Fig. 4. These results suggest moderate discriminative ability and reasonable performance considering the class imbalance in our dataset. The calibration plot showed good alignment between predicted probabilities and observed outcomes. Detailed information on threshold optimization and the calibration plot is provided in Supplementary Material 8. To evaluate the PKIP model’s performance across various pesticide categories, please consult Supplementary Material 9.

### Model-based risk stratification and survival analysis

**Fig. 5:**
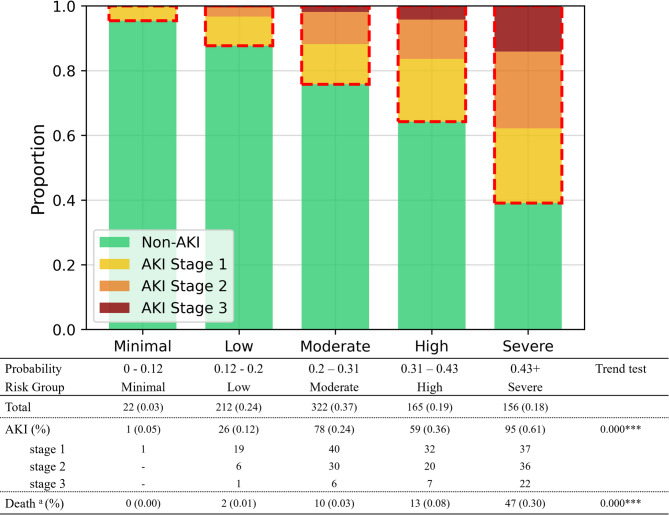
Risk stratification and outcomes using the Prediction of acute Kidney Injury in Pesticide intoxication (PKIP) model.

The PKIP model stratified patients into five risk groups based on predicted probabilities of AKI occurrence. The distribution of prediction probabilities is presented in Supplementary Material 10. The distribution of patients across these risk groups and their corresponding AKI and mortality are presented in Fig. 5. A significant trend was observed in both AKI occurrence (*p* < 0.001) and mortality (*p* < 0.001) across the risk groups, with higher risk groups showing progressively increased risks of AKI and death.

**Fig. 6:**
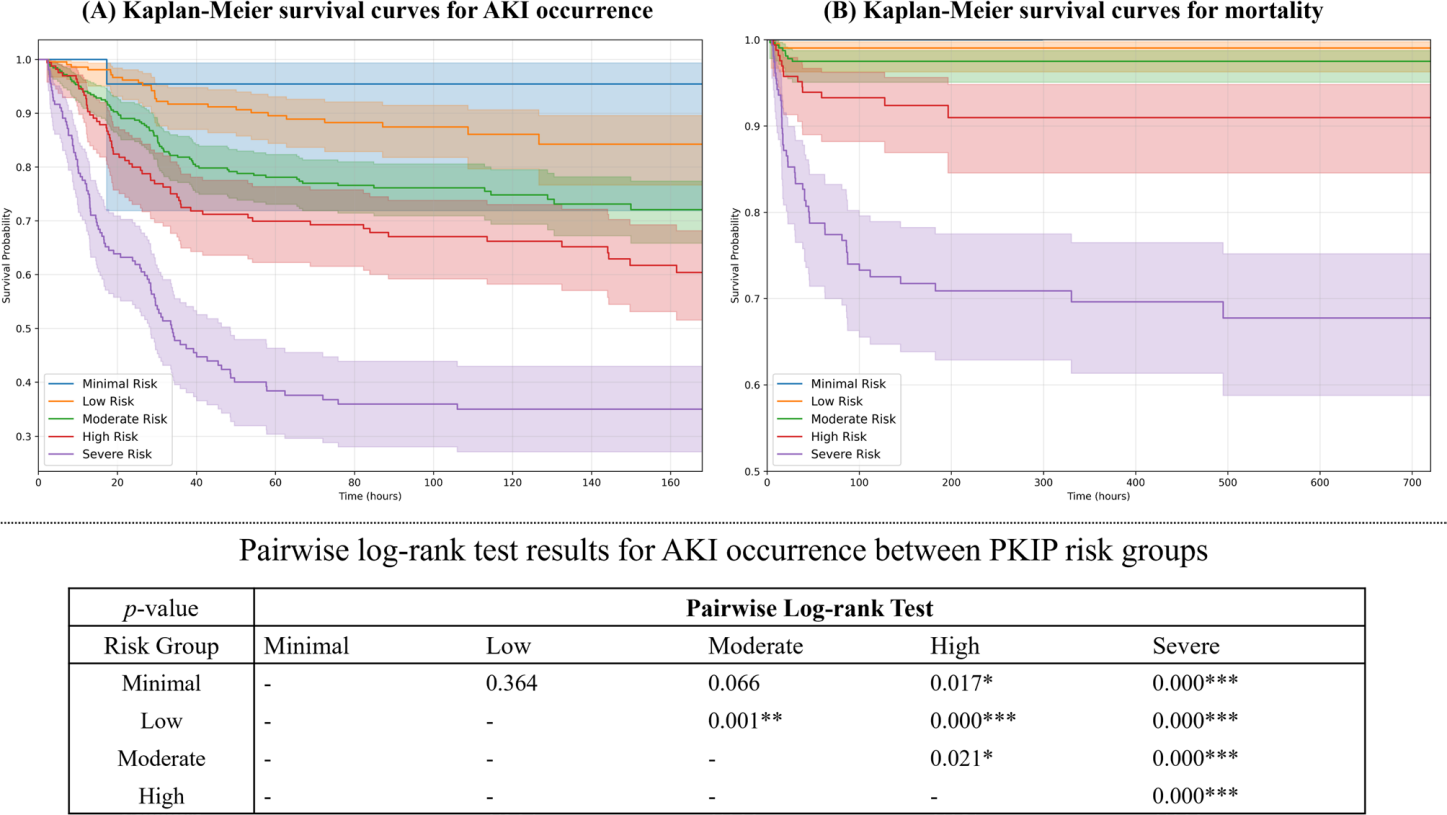
Risk stratification and survival analysis using the Prediction of acute Kidney Injury in Pesticide intoxication (PKIP) model.

Log-rank tests between risk groups revealed significant differences in survival curves, particularly between severe risk group (0.43 +) and all other groups (*p* < 0.001 for all comparisons). These results are presented in Fig. 6, suggesting that the PKIP model demonstrated moderate discriminative ability between patients with different levels of risk for adverse outcomes.

### Comparison with APACHE II score

To evaluate whether identifying patients at high risk of AKI could help in recognizing those with poor prognosis, we compared our developed AKI prediction model to the APACHE II score, a widely used severity assessment tool in intensive care units (ICU). Our PKIP model demonstrated superior performance in identifying trends for both AKI occurrence and mortality. While the APACHE II showed some discrimination ability, the trends were less pronounced (AKI: *p* = 0.091, Death: *p* = 0.205) compared to our model. For detailed information on the APACHE II score distribution and AKI and mortality distribution across risk groups, please refer to Supplementary Material 11. Additionally, survival analysis across score intervals can be found in Supplementary Materials 12 and 13.

Comparative performance metrics further support the comparative advantage of our model in risk stratification. For AKI prediction, our model achieved higher AUROC (0.720 vs 0.673) and AUPRC (0.513 vs 0.459) compared to the APACHE II. Similar improvements were observed for mortality prediction (AUROC: 0.839 vs 0.829; AUPRC: 0.421 vs 0.331). A detailed comparison of the results is presented in Supplementary Table 13.

### Hazard ratios and forest plots

**Fig. 7:**
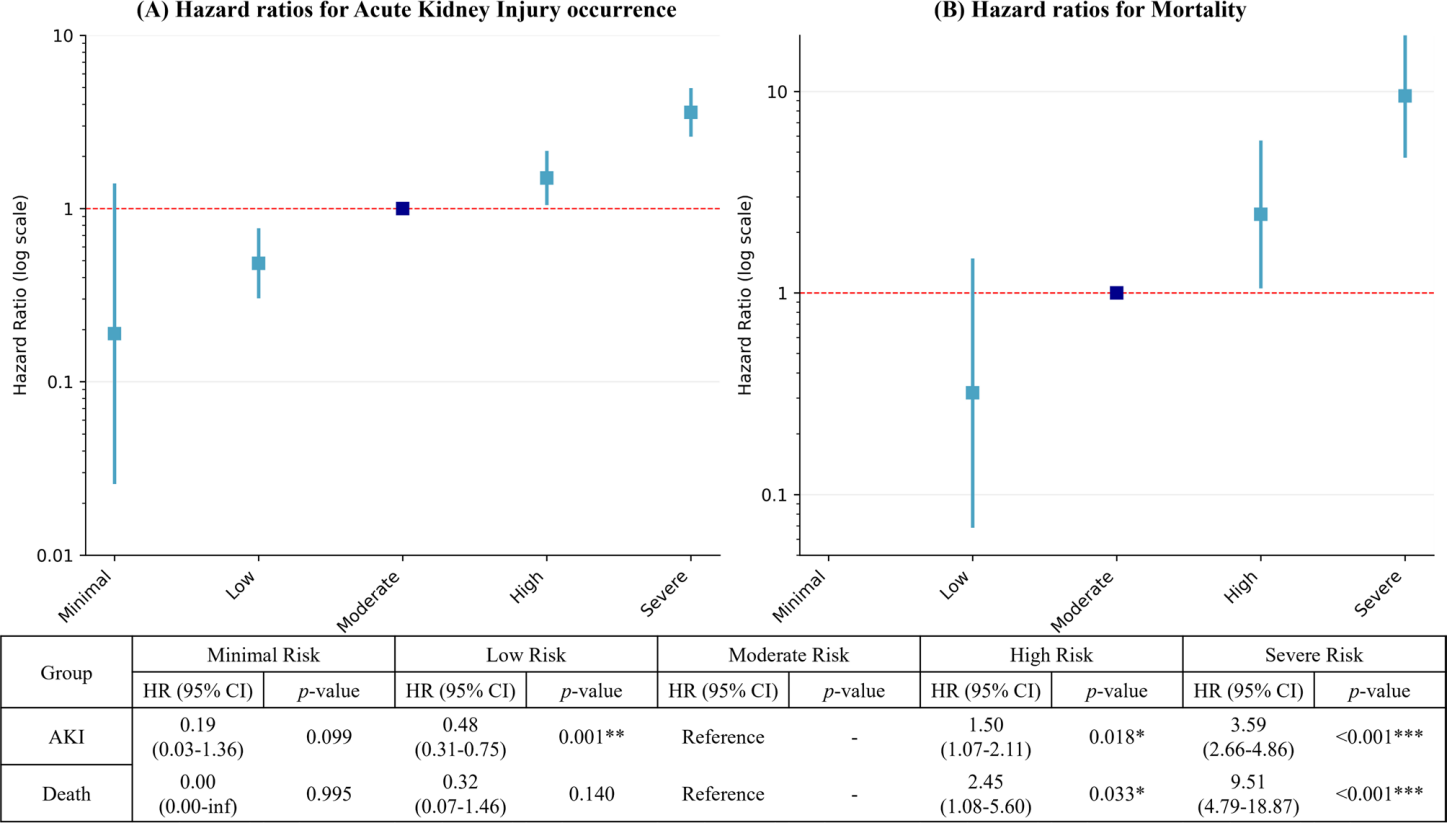
Hazard ratios for Acute Kidney Injury occurrence and mortality across the Prediction of acute Kidney Injury in Pesticide intoxication (PKIP) risk groups.

Figure 7 illustrates HR for AKI and mortality across different risk groups. Moderate risk group serves as the reference. For AKI, there was a clear gradient of risk, with severe risk group showing the highest HR of 3.59 (95% CI: 2.66–4.86, *p* < 0.001). The mortality risk followed a similar pattern, with severe risk having a substantially increased HR of 9.51 (95% CI: 4.79–18.87, *p* < 0.001) compared to the reference group.

## Discussion

Our study supports the applicability of KDIGO criteria for AKI identification in pesticide poisoning cases. Patients identified with AKI or higher AKI stages based on KDIGO criteria showed a significantly increased mortality risk compared to those without AKI or with lower stages. We developed an early AKI prediction model, PKIP, by strictly applying these KDIGO criteria. The model showed moderate performance with an AUROC of 0.720 (0.692–0.747) and an AUPRC of 0.513 (0.464–0.563).

The occurrence of AKI is significantly associated with adverse clinical outcomes, including prolonged ICU stay and increased mortality, presenting an elevated risk particularly in pesticide poisoning patients^24^. Early consultation with a nephrologist is essential prior to AKI development due to the necessity of considering multiple factors^25^. However, predicting AKI in advance remains challenging, especially in pesticide poisoning cases, as it requires the consideration of specific pesticide characteristics and the patient’s overall condition—a complex task for clinicians with limited experience^26^.

Recent studies have developed AKI prediction models across diverse clinical settings, including emergency departments, intensive care units, and general wards, as well as in specific scenarios such as surgery or exposure to nephrotoxic medications^27–31^. This emphasis on early prediction rather than treatment reflects a current clinical reality: there is currently no approved pharmacological therapy to prevent, treat, or enhance recovery from established AKI^32,33^. Given that recovery after established AKI is typically challenging, current research efforts focus predominantly on prevention strategies and early intervention before the onset of irreversible kidney damage ^32,34^.

These early AKI prediction models serve two critical clinical functions ^35,36^. First, they enable prevention-oriented treatment strategies, including optimization of volume status and hemodynamics, avoidance of nephrotoxic agents, and intensive input/output monitoring. Second, they function as clinical decision support tools, assisting with disposition decisions, patient prioritization, and treatment triage. By identifying patients at high risk for AKI early, these models can guide preventive management and interventions aimed at minimizing progression to severe AKI.

In terms of practical clinical application, the PKIP model is designed for integration into the early management workflow of acute pesticide poisoning patients in the emergency department. The 14 variables required by the model are all obtainable from routine initial evaluation within the first 2 h of presentation, without requiring additional specialized biomarkers. Once these variables are entered into the web-based PKIP tool, the model generates a predicted probability of AKI occurrence and stratifies patients into five risk categories, enabling graded clinical responses tailored to individual risk levels. For patients in the minimal or low-risk categories, standard monitoring with periodic renal function assessment may be sufficient. In contrast, those identified as high or severe-risk may benefit from more intensive preventive strategies, including optimization of fluid management, avoidance of nephrotoxic medications, closer monitoring of urine output and serum creatinine, and early nephrology consultation in a higher-acuity care setting^25,37^. This graded approach is consistent with the consensus framework proposed by the 27th Acute Disease Quality Initiative workgroup, which recommends leveraging digital health tools for risk stratification and tailored surveillance across the AKI continuum^38^. Similar web-based AKI prediction calculators have demonstrated feasibility and clinical utility in other settings, such as postoperative care and general hospitalized populations^29,31^. The PKIP tool may be particularly valuable in settings where clinical experience with pesticide-induced AKI is limited, by providing an objective, quantitative risk estimates that supplements clinical judgment and facilitates more standardized, risk-stratified care^26^.

Moreover, research focused on pesticide poisoning patients remains limited, and studies targeting specific pesticides restrict the general applicability of existing models in clinical practice^14,39,40^. This gap underscores the need for specialized prediction models tailored to the unique pathophysiology and clinical characteristics of pesticide-induced kidney injury.

Our study developed the PKIP model for predicting AKI occurrence in pesticide poisoning patients using 14 variables. The model classifies patients into risk groups to predict AKI occurrence risk. According to the PKIP model, the AKI incidence occurrence in the severe risk group was 61%, compared to 5% in the minimal risk group, representing an approximately 12-fold higher risk. The severity of AKI also differed significantly between risk groups. In the severe risk group, 23.2% of AKI patients had stage 3 AKI, compared to 7.7% in the moderate risk group and 3.8% in the low-risk group. Mortality also showed significant differences, reaching 30% in the severe risk group versus 0% in the minimal risk group.

Various scoring systems (e.g., APACHE II, SOFA, SAPS) have been used to predict prognosis^41–44^. However, general scoring systems should be validated for specific outcomes in the target population. The APACHE II score is one of the most widely used predictive tools for critically ill patients. Its scores are derived from the worst values within the initial 24 h of ICU admission. While commonly used, the calculation requires numerous physiological variables. In contrast, our PKIP model uses only 14 variables, making calculation simpler, and demonstrates better risk stratification performance in acute pesticide poisoning patients. PKIP is specifically tailored for this population, allowing clinically meaningful stratification of AKI and mortality risk.

Our study results show that when comparing mortality based on AKI occurrence, patients with AKI had a mortality of 16.6%, significantly higher than the 4.7% for patients without AKI. This suggests that early identification of patients at risk for AKI may help inform risk stratification and clinical decision-making, although its direct impact on mortality requires prospective validation^45^.Therefore, early prediction of AKI through the PKIP model may facilitate the consideration of preventive management strategies such as adequate hydration, antidote administration, UO maintenance, and urine alkalization, with the potential to improve patient prognosis^37^.

To ensure clinically reliable labeling and minimize false-positive AKI assignments, we constructed a comprehensive cohort during model development. This approach included two situations: (1) patients who died within 168 h without meeting AKI criteria (early censored deaths), who were labeled as non-AKI; and (2) cases with ambiguous AKI driven by transient sCr changes. The ambiguous AKI group (60 of 259 AKI cases) consisted of patients classified as AKI only when a transient decrease in sCr within the first 2 h of admission was accepted as a possible baseline. These patterns may indicate recovery from pre-admission AKI (consistent with the extended KDIGO)^46^ or short-term fluctuations caused by initial fluid resuscitation rather than true kidney injury^47,48^. Although their 168-h sCr variability was similar to the strict AKI group, their mortality (6.67%) was much closer to the non-AKI group (4.69%), indicating prognostic discordance and suggesting that many represent false positives. When these ambiguous cases were excluded and early-censored deaths were appropriately handled, the PKIP model’s performance improved from AUROC 0.7171 and AUPRC 0.5041 to AUROC 0.7550 and AUPRC 0.5262. Further details are provided in Supplementary Materials 14 and 15.

However, our study has several limitations. First, we utilized the KDIGO criteria for defining AKI. While KDIGO is widely used globally, future studies may need to establish criteria specifically tailored to pesticide poisoning. The application of KDIGO criteria to patients with acute pesticide poisoning presents important constraints, particularly in determining a reliable baseline serum creatinine at presentation and capturing rapid, transient changes in renal function. These factors may have resulted in misclassification of AKI status in some patients and could have influenced both the estimated incidence of AKI and the predictive performance of the model. Furthermore, accurate UO measurement is challenging due to clinical instability. Also, initial treatments such as fluid management or gastric lavage can temporarily influence fluid balance. Despite these challenges, we employed the KDIGO definition to ensure reproducibility and comparability with prior studies, while attempting to mitigate baseline-related bias through dynamic baseline creatinine definitions.

Second, our study was conducted at a single institution with a predominantly Korean population. Although our sample size is substantial for pesticide poisoning studies, it remains relatively small compared to other machine learning studies. Additionally, the mechanisms of pesticide-induced renal injury vary across different pesticide types. However, developing separate models for each pesticide type would risk overfitting. Moreover, research on the specific pathophysiology of AKI for individual agents remains scarce^49–52^. To address this, we employed a data-driven approach, classifying pesticides according to their clinical toxicity profiles and including pesticide category as a model feature. Accordingly, the transportability of the PKIP model to other healthcare systems, ethnic populations, and pesticide exposure patterns should be interpreted with caution. Future research should involve multi-ethnic, multi-center studies to validate the PKIP model across diverse populations.

Third, while AKI is consistently associated with increased mortality, whether AKI serves as a marker of severity, or a direct mediator of death may vary depending on patient characteristics and toxic agents. Our study focused on the prognostic association between AKI and mortality; however, the retrospective observational design does not allow causal inference regarding the role of AKI as a direct mediator of death^10,53–55^. However, prognosis extends beyond mortality, encompassing outcomes like CKD progression and quality of life. Future studies should comprehensively evaluate these diverse outcomes.

Taken together, these limitations suggest that the reported model performance and prognostic associations should be interpreted cautiously and primarily as hypothesis-generating rather than definitive evidence. Future studies should therefore focus on prospective, multi-center validation of the PKIP model across diverse populations and pesticide exposure profiles. In addition, alternative or complementary AKI labeling strategies and staged risk stratification frameworks should be explored to improve diagnostic robustness and clinical interpretability. Finally, prospective implementation studies are needed to determine whether early AKI risk prediction meaningfully influences clinical decision-making and patient-centered outcomes.

## Conclusion

This study demonstrates that KDIGO-defined AKI is a significant prognostic factor for mortality in patients with acute pesticide poisoning, with AKI patients showing a substantially higher mortality rate (16.6%) compared to non-AKI patients (4.7%). Risk stratification using PKIP showed a significant gradient of adverse outcomes, with the severe-risk group exhibiting approximately 12-fold higher AKI incidence and 30% mortality compared to the minimal-risk group. The accompanying web-based tool provides a practical instrument for individualized AKI risk assessment at the bedside, potentially supporting early clinical decision-making, including risk-appropriate monitoring intensity, preventive fluid management, and timely nephrology consultation. However, these findings should be interpreted with caution given the single-center, retrospective design and the moderate discrimination level of the model. Future research should prioritize prospective, multi-center external validation across diverse populations, ethnic groups, and pesticide exposure profiles.

## Supplementary Information

Below is the link to the electronic supplementary material.

Supplementary Material 1

Supplementary Material 2

## Data Availability

The data that support the findings of this study contain potentially identifiable patient information and are not publicly available due to institutional and ethical restrictions, which prohibit public sharing of individual-level data even in de-identified form. Nevertheless, the data from this study can be acquired upon reasonable request from the corresponding author and subject to approval by the institutional review board and the participating institution.

## Abbreviations

ABG: Arterial blood gas
AG: Anion gap
AKI: Acute kidney injury
ALP: Alkaline phosphatase
AUROC: Area under the precision-recall curve
AUROC: Area under the receiver operating characteristic curve
BMI: Body mass index
CAT: CatBoost
CI: Confidence interval
CKD: Chronic kidney disease
EHR: Electronic health records
GCS: Glasgow Coma Scale
Hb: Hemoglobin
HR: Hazard ratio
ICU: Intensive care unit
KDIGO: Kidney Disease: Improving Global Outcomes
LASSO: Least absolute shrinkage and selection operator
PKIP: Prediction of acute Kidney Injury in Pesticide intoxication
RBC: Red blood cell
sCr: Serum creatinine
UO: Urine output
WBC: White blood cell
PKIP: Prediction of acute Kidney Injury in Pesticide intoxication
