# Supplementary material for: Prediction of acute kidney injury in patients with acute pesticide poisoning using the PKIP score
Source: Sci Rep. 2026 Mar 26;16:15086. doi: 10.1038/s41598-026-41334-4 (PMC13172389; doi:10.1038/s41598-026-41334-4)
Supplement: Supplementary file 1 — Supplementary Material 1 [file 41598_2026_41334_MOESM1_ESM.docx]

**Supplementary Material 1. Proportion of Missing Data for Variables in the Study Population**

| **Features** | **Missing Values** | **Missing Percentage (%)** |
| --- | --- | --- |
| Age, years | 0 | 0.0 |
| Sex, male (%) | 0 | 0.0 |
| Body Mass Index, kg/m^2^ | 68 | 7.8 |
| Alcohol history, yes (%) | 14 | 1.6 |
| Diabetes, present (%) | 3 | 0.3 |
| Hypertension, present (%) | 3 | 0.3 |
| Lung disease, present (%) | 3 | 0.3 |
| Cardiovascular disease, present (%) | 3 | 0.3 |
| Chronic kidney disease, present (%) | 0 | 0.0 |
| Neuropsychiatry disease, present (%) | 3 | 0.3 |
| Hypoxemia, pO2 ≤ 60 (%) | 4 | 0.5 |
| Hypercapnea, pCO^2^ > 60 (%) | 4 | 0.5 |
| Glasgow Coma Scale score | 3 | 0.3 |
| APACHE II score | 3 | 0.3 |
| Pesticide category ^a^ | 0 | 0.0 |
| Amount of ingestion | 0 | 0.0 |
| Systolic blood pressure, mmHg | 0 | 0.0 |
| Pulse rate, beats/min | 0 | 0.0 |
| Respiratory rate, breaths/min | 0 | 0.0 |
| Body temperature, ˚C | 0 | 0.0 |
| Hemoglobin, g/dL | 0 | 0.0 |
| White Blood Cell count, 10³/μL | 0 | 0.0 |
| Platelet count, 10³/μL | 0 | 0.0 |
| Albumin, mg/dL | 0 | 0.0 |
| Glucose, mg/dL | 0 | 0.0 |
| Creatinine, mg/dL | 0 | 0.0 |
| Uric Acid, mg/dL | 0 | 0.0 |
| Phosphate, mg/dL | 0 | 0.0 |
| Sodium, mmol/L | 0 | 0.0 |
| Potassium, mmol/L | 0 | 0.0 |
| Calcium, mg/dL | 0 | 0.0 |
| C-Reactive Protein, mg/L | 0 | 0.0 |
| Arterial pH | 4 | 0.5 |
| Partial pressure of carbon dioxide, mmHg | 4 | 0.5 |
| Partial pressure of oxygen, mmHg | 4 | 0.5 |
| Bicarbonate , mmol/L | 4 | 0.5 |
| Anion gap, mmol/L | 43 | 4.9 |
| Alkaline phosphatase, U/L | 0 | 0.0 |
| Aspartate transaminase, U/L | 0 | 0.0 |
| Blood urea nitrogen, mg/dL | 0 | 0.0 |
| Urine specific gravity | 0 | 0.0 |
| Triglyceride, mg/dL | 0 | 0.0 |
| Total bilirubin, mg/dL | 0 | 0.0 |
| Total cholesterol, mg/dL | 0 | 0.0 |
| Total protein, g/dL | 0 | 0.0 |
| Lactate level, mmol/L | 21 | 2.4 |
| Activated Partial Thromboplastin Time, seconds | 1 | 0.1 |
| Prothrombin Time - International Normalized Ratio | 0 | 0.0 |
| Urine Red Blood Cell, cells | 0 | 0.0 |
| Urine protein, mg/dL | 0 | 0.0 |
| Acute Kidney Injury | 0 | 0.0 |
| Death ^b^, yes (%) | 0 | 0.0 |

Data are presented as the number of missing values count and the percentage of missing data (%).

^a^ Pesticides category include Glufosinate, Glyphosate, Organophosphate or Carbamate, Pyrethroid and Other pesticides include acetanilide, acetylaniline, alryoxylcarboxide, amide, anilin, arsenic, phenopropionate, benzohydrazide, benzoate, chlorfenapyr, chloroacetamide, chloronicotinyl, diamide, diazine, dinitroaniline, endosulfan, fungicide, insect growth regulator, lambda cyhalothrin, neonicotinoid, niacin, oxadiazole, phenoxy, pyrol, sulfonylurea, sulfoximine, sulfuryl fluoride, tetramic acid, tetrazolium oxide, and unknown pesticides..

^b^ Death refers to all-cause mortality.

Supplementary Material 2. Ranges of hyperparameters

| **Model** | **Hyperparameter** |
| --- | --- |
| Logistic Regression | {max_iter': [10000], 'C': [0.001, 0.01, 0.1, 1.0, 10.0]} |
| Random Forest | {'max_depth': [None] + list(range(5, 10)), 'n_estimators': [50, 100, 300], 'min_samples_split': [2, 3, 4], 'min_samples_leaf': [1, 2, 3]} |
| eXtreme Gradient Boost | {'n_estimators': [50, 100, 300], 'max_depth': list(range(5, 10)), 'learning_rate': [0.001, 0.003, 0.01, 0.03], 'subsample': [0.7, 0.8, 0.9, 1], 'colsample_bytree': [0.7, 0.8, 0.9, 1]} |
| Light Gradient Boost | {'n_estimators': [50, 100, 300], 'max_depth': [-1] + list(range(5, 10)), 'learning_rate': [0.001, 0.003, 0.01, 0.03]} |
| Categorical Boost | {'iterations': [50, 100, 300], 'depth': list(range(5, 10)), 'learning_rate': [0.001, 0.003, 0.009, 0.01, 0.03], 'subsample': [0.7, 0.8, 0.9, 1], 'l2_leaf_reg': [1, 2, 3, 5]} |
| Support Vector Machine | {'C': [0.001, 0.01, 0.1, 1.0, 10.0], 'kernel': ['linear', 'rbf'], 'gamma': ['scale', 'auto']} |

**
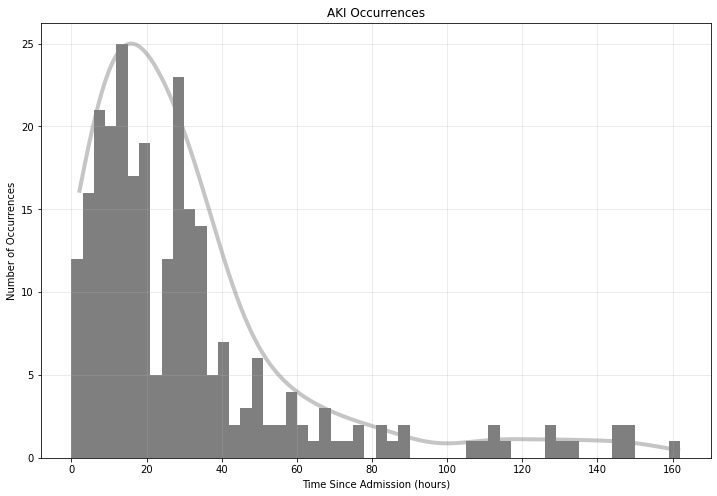
**

Supplementary Material 3. Timeline of Acute Kidney Injury incident rate

**Supplementary Material 4. Univariate COX Regression Analysis**

| **Variables** | **HR (95% CI)** | ***p*-value** |
| --- | --- | --- |
| Age, year | 1.02 (1.01-1.02) | 0.000******* |
| Sex, male versus female | 1.21 (0.92-1.58) | 0.175 |
| Body Mass Index, kg/m^2^ | 1.05 (1.01-1.09) | 0.006** |
| Alcohol history, yes versus no | 0.86 (0.67-1.12) | 0.270 |
| Diabetes, present versus absent | 0.97 (0.70-1.35) | 0.860 |
| Hypertension, present versus absent | 1.02 (0.78-1.33) | 0.880 |
| Pulmonary, present versus absent | 1.12 (0.74-1.71) | 0.589 |
| Cardiovascular disease, present versus absent | 0.99 (0.56-1.73) | 0.962 |
| Chronic kidney disease, present versus absent | 3.41 (1.40-8.29) | 0.007** |
| Neuropsychiatry disease, present versus absent | 1.09 (0.80-1.49) | 0.582 |
| Hypoxemia, yes versus no | 1.50 (1.02-2.22) | 0.041* |
| Hypercapnea, yes versus no | 0.91 (0.69-1.21) | 0.536 |
| Glasgow Coma Scale score | 0.89 (0.86-0.92) | 0.000*** |
| APACHE II score | 1.07 (1.06-1.09) | 0.000*** |
| **Pesticide category** |  |  |
| Organophosphate, Carbamate  or Glufosinate versus | 1.68 (1.30-2.17) | 0.000*** |
| Other pesticides ^a^ (e.g., pyrethroid) | Reference |  |
| **Amount of ingestion, cc** |  |  |
| ≤ 100 | Reference |  |
| 100 - 300 | 1.44 (1.06-1.97) | 0.020* |
| > 300 | 1.54 (1.04-2.29) | 0.031* |
| Unknown | 1.53 (0.99-2.36) | 0.055 |
| Systolic blood pressure, mmHg | 1.00 (0.99-1.00) | 0.847 |
| Pulse rate, beats/min | 1.01 (1.01-1.02) | 0.000*** |
| Respiratory rate, breaths/min | 1.00 (0.96-1.04) | 0.936 |
| Body temperature, ˚C | 0.93 (0.90-0.97) | 0.000*** |
| Hemoglobin, g/dL | 1.08 (1.01-1.16) | 0.030 |
| White Blood Cell count, 10³/μL | 1.04 (1.02-1.06) | 0.000*** |
| Platelet count, 10³/μL | 1.00 (1.00-1.00) | 0.256 |
| Albumin, mg/dL | 0.98 (0.76-1.25) | 0.854 |
| Glucose, mg/dL | 1.00 (1.00-1.00) | 0.001** |
| Creatinine, mg/dL | 2.93 (2.03-4.22) | 0.000*** |
| Uric Acid, mg/dL | 1.10 (1.03-1.18) | 0.006** |
| Phosphate, mg/dL | 1.20 (1.07-1.33) | 0.001** |
| Sodium, mmol/L | 1.02 (0.99-1.06) | 0.264 |
| Potassium, mmol/L | 1.03 (0.84-1.27) | 0.758 |
| Calcium, mg/dL | 0.97 (0.82-1.14) | 0.687 |
| C-Reactive Protein, mg/L | 1.00 (1.00-1.01) | 0.578 |
| Arterial pH (per 0.01 increase) | 0.97 (0.95-0.98) | 0.000*** |
| Partial pressure of carbon dioxide, mmHg | 1.01 (1.00-1.02) | 0.219 |
| Partial pressure of oxygen, mmHg | 1.00 (1.00-1.01) | 0.021* |
| Bicarbonate , mmol/L | 0.93 (0.91-0.96) | 0.000*** |
| Anion gap, mmol/L | 1.08 (1.05-1.10) | 0.000*** |
| Alkaline phosphatase, U/L | 1.01 (1.00-1.01) | 0.002** |
| Aspartate transaminase, U/L | 1.00 (1.00-1.00) | 0.027* |
| Blood urea nitrogen, mg/dL | 1.03 (1.01-1.05) | 0.001* |
| Urine specific gravity | 1.05 (0.90-1.23) | 0.531 |
| Triglyceride, mg/dL | 1.00 (1.00-1.00) | 0.200 |
| Total bilirubin, mg/dL | 1.42 (1.07-1.90) | 0.017* |
| Total cholesterol, mg/dL | 1.00 (1.00-1.00) | 0.812 |
| Total protein, g/dL | 1.14 (0.94-1.39) | 0.170 |
| Lactate level, mmol/L | 1.12 (1.07-1.16) | 0.000*** |
| Activated Partial Thromboplastin Time, seconds | 1.00 (1.00-1.01) | 0.380 |
| Prothrombin Time - International Normalized Ratio | 1.02 (0.92-1.13) | 0.727 |
| **Urine Red Blood Cell, cells** |  |  |
| <1 | Reference |  |
| ≥1 | 1.43 (1.12-1.84) | 0.005** |
| **Urine protein, mg/dL** |  |  |
| Negative | Reference |  |
| Trace | 1.64 (1.28-2.10) | 0.000*** |
| ≥1 | 2.20 (1.49-3.23) | 0.000*** |

**Abbreviations:** HR, hazard ratio; CI, confidence interval; APACHE, Acute Physiology and Chronic Health Evaluation

Data are represented as hazard ratios with 95% confidence intervals.

^a^ Other pesticides include acetanilide, acetylaniline, alryoxylcarboxide, amide, anilin, arsenic, phenopropionate, benzohydrazide, benzoate, chlorfenapyr, chloroacetamide, chloronicotinyl, diamide, diazine, dinitroaniline, endosulfan, fungicide, insect growth regulator, lambda cyhalothrin, neonicotinoid, niacin, oxadiazole, phenoxy, pyrol, sulfonylurea, sulfoximine, sulfuryl fluoride, tetramic acid, tetrazolium oxide, and unknown pesticides.

* indicates *p*-value < 0.05

** indicates *p*-value < 0.01

*** indicates *p*-value < 0.001

**Supplementary Material 5. Univariate COX regression analysis based on Pesticide groups**

| **Features** | **Other pesticides ^a^ (e.g., pyrethroid)** | | **Organophosphate, Carbamate or Glufosinate** | |
| --- | --- | --- | --- | --- |
|  | **HR (95% CI)** | ***p*-value** | **HR (95% CI)** | ***p* -value** |
| Age, year | 1.01 (1.00-1.03) | 0.026* | 1.02 (1.01-1.03) | 0.003** |
| Sex, male versus female | 1.17 (0.79-1.73) | 0.427 | 1.32 (0.90-1.93) | 0.156 |
| Body Mass Index, kg/m^2^ | 1.06 (1.01-1.12) | 0.027* | 1.04 (0.99-1.10) | 0.111 |
| Alcohol history, yes versus no | 0.85 (0.59-1.23) | 0.394 | 0.90 (0.63-1.30) | 0.591 |
| Diabetes, present versus absent | 0.98 (0.62-1.56) | 0.934 | 0.98 (0.61-1.58) | 0.949 |
| Hypertension, present versus absent | 0.91 (0.62-1.33) | 0.619 | 1.14 (0.79-1.66) | 0.472 |
| Pulmonary, present versus absent | 1.18 (0.66-2.10) | 0.578 | 1.10 (0.59-2.05) | 0.766 |
| Cardiovascular disease, present versus absent | 1.10 (0.48-2.50) | 0.825 | 0.84 (0.39-1.80) | 0.648 |
| Chronic kidney disease, present versus absent | 2.03 (0.28-14.58) | 0.480 | 3.37 (1.24-9.19) | 0.018* |
| Neuropsychiatry disease, present versus absent | 0.87 (0.53-1.42) | 0.576 | 1.17 (0.78-1.75) | 0.443 |
| Hypoxemia, yes versus no | 1.80 (1.06-3.06) | 0.029* | 1.23 (0.69-2.19) | 0.483 |
| Hypercapnea, yes versus no | 0.80 (0.52-1.22) | 0.291 | 0.97 (0.66-1.43) | 0.875 |
| Glasgow Coma Scale score | 0.89 (0.85-0.93) | 0.000*** | 0.90 (0.86-0.94) | 0.000*** |
| APACHE II score | 1.07 (1.04-1.09) | 0.000*** | 1.07 (1.05-1.10) | 0.000*** |
| **Amount of ingestion, cc** |  |  |  |  |
| ≤ 100 | Reference |  | Reference |  |
| 100 – 300 | 1.32 (0.86-2.05) | 0.206 | 1.33 (0.84-2.09) | 0.221 |
| > 300 | 1.51 (0.85-2.70) | 0.161 | 1.31 (0.75-2.29) | 0.336 |
| Unknown | 1.76 (1.01-3.06) | 0.047* | 1.20 (0.60-2.40) | 0.610 |
| Systolic blood pressure, mmHg | 0.99 (0.98-1.00) | 0.040* | 1.01 (1.00-1.01) | 0.157 |
| Pulse rate, beats/min | 1.01 (1.00-1.02) | 0.084 | 1.02 (1.01-1.03) | 0.000*** |
| Respiratory rate, breaths/min | 1.00 (0.94-1.06) | 0.960 | 1.00 (0.95-1.05) | 0.959 |
| Body temperature, ˚C | 0.93 (0.89-0.97) | 0.001** | 0.77 (0.61-0.97) | 0.024* |
| Hemoglobin, g/dL | 1.01 (0.92-1.12) | 0.820 | 1.16 (1.06-1.28) | 0.002** |
| White Blood Cell count, 10³/μL | 1.03 (1.00-1.06) | 0.065 | 1.05 (1.02-1.08) | 0.000*** |
| Platelet count, 10³/μL | 1.00 (1.00-1.00) | 0.118 | 1.00 (1.00-1.00) | 0.768 |
| Albumin, mg/dL | 0.90 (0.63-1.28) | 0.544 | 1.08 (0.77-1.51) | 0.664 |
| Glucose, mg/dL | 1.00 (1.00-1.00) | 0.189 | 1.00 (1.00-1.01) | 0.002** |
| Creatinine, mg/dL | 2.75 (1.65-4.58) | 0.000*** | 3.41 (1.99-5.84) | 0.000*** |
| Uric Acid, mg/dL | 1.11 (1.00-1.23) | 0.047* | 1.12 (1.02-1.23) | 0.014* |
| Phosphate, mg/dL | 1.15 (0.99-1.34) | 0.075 | 1.25 (1.07-1.46) | 0.005** |
| Sodium, mmol/L | 1.03 (0.98-1.09) | 0.208 | 1.02 (0.97-1.07) | 0.381 |
| Potassium, mmol/L | 1.27 (1.01-1.59) | 0.043* | 0.84 (0.60-1.18) | 0.324 |
| Calcium, mg/dL | 0.94 (0.74-1.18) | 0.588 | 1.08 (0.83-1.39) | 0.569 |
| C-Reactive Protein, mg/L | 1.00 (0.99-1.01) | 0.479 | 1.00 (0.99-1.01) | 0.731 |
| Arterial pH (per 0.01 increase) | 0.96 (0.94-0.98) | 0.000*** | 0.97 (0.95-0.99) | 0.009** |
| Partial pressure of carbon dioxide, mmHg | 1.00 (0.97-1.02) | 0.760 | 1.01 (0.99-1.02) | 0.319 |
| Partial pressure of oxygen, mmHg | 1.00 (1.00-1.01) | 0.135 | 1.00 (1.00-1.00) | 0.317 |
| Bicarbonate , mmol/L | 0.91 (0.88-0.95) | 0.000*** | 0.95 (0.92-0.99) | 0.017 |
| Anion gap, mmol/L | 1.09 (1.05-1.12) | 0.000*** | 1.08 (1.04-1.11) | 0.000*** |
| Alkaline phosphatase, U/L | 1.01 (1.00-1.01) | 0.090 | 1.01 (1.00-1.01) | 0.011* |
| Aspartate transaminase, U/L | 1.00 (1.00-1.00) | 0.363 | 1.00 (1.00-1.01) | 0.015* |
| Blood urea nitrogen, mg/dL | 1.03 (1.00-1.06) | 0.036* | 1.03 (1.00-1.06) | 0.033* |
| Urine specific gravity | 1.11 (0.89-1.39) | 0.352 | 0.93 (0.75-1.16) | 0.540 |
| Triglyceride, mg/dL | 1.00 (1.00-1.00) | 0.886 | 1.00 (1.00-1.00) | 0.061 |
| Total bilirubin, mg/dL | 1.16 (0.75-1.79) | 0.498 | 1.81 (1.24-2.64) | 0.002** |
| Total cholesterol, mg/dL | 1.00 (0.99-1.00) | 0.541 | 1.00 (1.00-1.00) | 0.720 |
| Total protein, g/dL | 1.00 (0.75-1.34) | 0.993 | 1.30 (1.01-1.66) | 0.041* |
| Lactate level, mmol/L | 1.13 (1.08-1.20) | 0.000*** | 1.13 (1.05-1.21) | 0.001** |
| Activated Partial Thromboplastin Time, seconds | 1.01 (1.00-1.01) | 0.013* | 0.99 (0.98-1.01) | 0.489 |
| Prothrombin Time - International Normalized Ratio | 0.03 (0.00-0.30) | 0.002** | 0.96 (0.80-1.15) | 0.654 |
| **Urine Red Blood Cell, cells/HPF** |  |  |  |  |
| <1 | Reference |  | Reference |  |
| ≥1 | 1.32 (0.91-1.92) | 0.148 | 1.25 (0.84-1.87) | 0.271 |
| **Urine erythrocyte** |  |  |  |  |
| Negative | Reference |  | Reference |  |
| Trace | 1.56 (1.04-2.35) | 0.032* | 1.32 (0.86-2.02) | 0.199 |
| ≥1 | 1.34 (0.81-2.21) | 0.251 | 1.23 (0.78-1.95) | 0.372 |
| **Urine protein** |  |  |  |  |
| Negative | Reference |  | Reference |  |
| Trace | 1.69 (1.15-2.48) | 0.008** | 1.24 (0.84-1.83) | 0.271 |
| ≥1 | 1.80 (0.94-3.45) | 0.077 | 2.11 (1.17-3.80) | 0.013* |

**Abbreviations:** HR, hazard ratio; CI, confidence interval.

Data are represented as hazard ratios with 95% confidence intervals.

^a^ Other pesticides includes acetanilide, acetylaniline, aryloxycarboxide, amide, anilin, arsenic, phenopropionate, benzohydrazide, benzoate, chlorfenapyr, chloroacetamide, chloronicotinyl, diamide, diazine, dinitroaniline, endosulfan, fungicide, insect growth regulator, lambda cyhalothrin, neonicotinoid, niacin, oxadiazole, phenoxy, pyrol, sulfonylurea, sulfoximine, sulfuryl fluoride, tetramic acid, tetrazolium oxide, and unknown pesticides.

* Indicates *p*-value < 0.05

** indicates *p*-value < 0.01

*** indicates *p*-value < 0.001

Supplementary Material 6. Selected Variables and Hyperparameters for Different Feature Selection Methods

| **Feature Selection** | **Total Selection ^a^** | **Clinical Selection ^b^** | **Stepwise Selection ^c^** | **LASSO Selection ^d^** |
| --- | --- | --- | --- | --- |
|  | **Binary feature** | **Binary feature** | **Binary feature** | **Binary feature** |
|  | Hypoxemia  Chronic kidney disease | Chronic kidney disease | Chronic kidney disease | Hypoxemia  Chronic kidney disease |
|  | **Numerical feature** | **Numerical feature** | **Numerical feature** | **Numerical feature** |
|  | Age  Body Mass Index  Pulse rate  Body temperature  Glasgow Coma Scale score  Arterial pH  Partial pressure of oxygen  Bicarbonate  Glucose  Creatinine  Uric Acid  Phosphate  White Blood Cell count  Hemoglobin  Anion gap  Alkaline phosphatase  Aspartate transaminase  Blood urea nitrogen  Total bilirubin  Lactate | Age  Body Mass Index  Pulse rate  Body temperature  Glasgow Coma Scale score  Arterial pH  Partial pressure of oxygen  Bicarbonate  Creatinine  Uric Acid  Phosphate  White Blood Cell count  Hemoglobin  Blood urea nitrogen  Lactate  Sodium  Potassium  Partial pressure of carbon dioxide  Sex  Diabetes  Hypertension  Systolic blood pressure  Respiratory rate  Platelet count  Albumin  C-Reactive Protein  Urine specific gravity | Age  Body Mass Index  Glasgow Coma Scale score  Bicarbonate  Hemoglobin, g/dL  Anion gap  Alkaline phosphatase | Age  Body Mass Index  Body temperature  Glasgow Coma Scale score  Bicarbonate  Phosphate  White Blood Cell count  Hemoglobin  Anion gap  Alkaline phosphatase |
|  | **Categorical feature** | **Categorical feature** | **Categorical feature** | **Categorical feature** |
|  | Amount of ingestion  Pesticide category  Urine erythrocyte  Urine protein  Urine Red Blood Cell | Amount of ingestion  Pesticide category  Urine protein | Pesticide category | Pesticide category  Urine Red Blood Cell |
| **Model** | **Hyperparameter tuning** | | | |
| Logistic Regression | {'C': 0.01, 'max_iter': 10000} | {'C': 0.1, 'max_iter': 10000} | {'C': 0.1, 'max_iter': 10000} | {'C': 10.0, 'max_iter': 10000} |
| Random Forest | {'max_depth': 6,'min_samples_leaf': 1,'min_samples_split': 4,'n_estimators': 50} | {'max_depth': 6,'min_samples_leaf': 1,'min_samples_split': 3,'n_estimators': 300} | {'max_depth': 5,'min_samples_leaf': 1,'min_samples_split': 4,'n_estimators': 50} | {'max_depth': 5,'min_samples_leaf': 2,'min_samples_split': 2,'n_estimators': 100} |
| eXtreme Gradient Boost | {'colsample_bytree': 0.8,'learning_rate': 0.003,'max_depth': 8,'n_estimators': 300,'subsample': 0.7} | {'colsample_bytree': 0.7, 'learning_rate': 0.03,'max_depth': 5,'n_estimators': 100,'subsample': 0.8} | {'colsample_bytree': 0.7,'learning_rate': 0.001,'max_depth': 5,'n_estimators': 300,'subsample': 0.8} | {'colsample_bytree': 0.7,'learning_rate': 0.003,'max_depth': 5,'n_estimators': 300,'subsample': 0.8} |
| Light Gradient Boost | {'learning_rate': 0.03, 'max_depth': 7, 'n_estimators': 100} | {'learning_rate': 0.03, 'max_depth': 5, 'n_estimators': 100} | {'learning_rate': 0.03, 'max_depth': 5, 'n_estimators': 100} | {'learning_rate': 0.03, 'max_depth': 5, 'n_estimators': 50} |
| **Categorical Boost** | **{'depth': 7, 'iterations': 50, 'l2_leaf_reg': 3, 'learning_rate': 0.009, 'subsample': 0.9}** | **{'depth': 7, 'iterations': 300, 'l2_leaf_reg': 1, 'learning_rate': 0.009, 'subsample': 1}** | **{'depth': 6,'iterations': 100,'l2_leaf_reg': 1,'learning_rate': 0.03,'subsample': 0.8}** | **{'depth': 5,'iterations': 300,'l2_leaf_reg': 3,'learning_rate': 0.01,'subsample': 1}** |
| Support Vector Machine | {'C': 1.0, 'gamma': 'scale', 'kernel': 'linear'} | {'C': 10.0, 'gamma': 'scale', 'kernel': 'linear'} | {'C': 0.1, 'gamma': 'scale', 'kernel': 'linear'} | {'C': 1.0, 'gamma': 'scale', 'kernel': 'linear'} |

LASSO, Least Absolute Shrinkage and Selection Operator

^a^ Total Selection refers to the full variable approach, which includes all potential predictive variables in the initial model to assess the contribution of each variable.

^b^ Clinical Selection refers to clinical expertise-based selection, which prioritizes variables deemed clinically important by nephrologists.

^c^ Stepwise Selection refers to combining forward and backward elimination methods to iteratively add or remove variables based on statistical significance.

^d^ LASSO Selection refers to Least Absolute Shrinkage and Selection Operator regression, which selects the most relevant variables while shrinking coefficients and preventing overfitting through regularization

**Supplementary Material 7. Comparison of Model Performance for Acute Kidney Injury Prediction Using Different Feature Selection Methods Across Six Machine Learning Models**

| **Training Set** | **Total Selection ^a^** | | **Clinical Selection ^b^** | | **Stepwise Selection ^c^** | | **LASSO Selection ^d^** | |
| --- | --- | --- | --- | --- | --- | --- | --- | --- |
|  | AUROC | **95% CI** | AUROC | **95% CI** | AUROC | **95% CI** | AUROC | **95% CI** |
| Logistic Regression | 0.690 | 0.649 - 0.730 | 0.673 | 0.634 - 0.712 | 0.698 | 0.658 - 0.738 | 0.706 | 0.667 - 0.745 |
| Random Forest | 0.698 | 0.665 - 0.731 | 0.676 | 0.642 - 0.709 | 0.714 | 0.673 - 0.755 | 0.709 | 0.663 - 0.755 |
| eXtreme Gradient Boost | 0.691 | 0.663 - 0.720 | 0.661 | 0.612 - 0.710 | 0.705 | 0.670 - 0.739 | 0.702 | 0.667 - 0.737 |
| Light Gradient Boost | 0.678 | 0.644 - 0.712 | 0.654 | 0.600 - 0.708 | 0.698 | 0.672 - 0.724 | 0.686 | 0.646 - 0.726 |
| **Categorical Boost** | **0.715** | **0.682 - 0.747** | **0.689** | **0.655 - 0.723** | **0.716** | **0.690 - 0.743** | **0.720** | **0.693 - 0.747** |
| Support Vector Machine | 0.694 | 0.649 - 0.739 | 0.662 | 0.631 - 0.692 | 0.693 | 0.661 - 0.725 | 0.700 | 0.652 - 0.748 |
|  | AUPRC | **95% CI** | AUPRC | **95% CI** | AUPRC | **95% CI** | AUPRC | **95% CI** |
| Logistic Regression | 0.486 | 0.441 - 0.531 | 0.463 | 0.409 - 0.517 | 0.509 | 0.422 - 0.596 | 0.507 | 0.432 - 0.582 |
| Random Forest | 0.484 | 0.433 - 0.536 | 0.472 | 0.437 - 0.506 | 0.519 | 0.456 - 0.583 | 0.524 | 0.441 - 0.608 |
| eXtreme Gradient Boost | 0.488 | 0.428 - 0.549 | 0.453 | 0.385 - 0.521 | 0.497 | 0.428 - 0.565 | 0.497 | 0.431 - 0.563 |
| Light Gradient Boost | 0.468 | 0.418 - 0.519 | 0.455 | 0.392 - 0.518 | 0.496 | 0.452 - 0.541 | 0.464 | 0.394 - 0.534 |
| **Categorical Boost** | **0.522** | **0.469 - 0.575** | **0.481** | **0.421 - 0.542** | **0.516** | **0.444 - 0.589** | **0.513** | **0.464 - 0.563** |
| Support Vector Machine | 0.500 | 0.416 - 0.584 | 0.457 | 0.423 - 0.490 | 0.500 | 0.428 - 0.573 | 0.514 | 0.416 - 0.612 |

**Abbreviations:** AUROC, Area Under the Receiver Operating Characteristic curve; AUPRC, Area Under the Precision-Recall Curve; CI, Confidence Interval.

^a^ Total Selection refers to the full variable approach, which includes all potential predictive variables in the initial model to assess the contribution of each variable.

^b^ Clinical Selection refers to clinical expertise-based selection, which prioritizes variables deemed clinically important by nephrologists.

^c^ Stepwise Selection refers to combining forward and backward elimination methods to iteratively add or remove variables based on statistical significance.

^d^ LASSO Selection refers to Least Absolute Shrinkage and Selection Operator regression, which selects the most relevant variables while shrinking coefficients and preventing overfitting through regularization

Supplementary Material 8. The Prediction of acute Kidney Injury in Pesticide intoxication (PKIP) model Evaluation Metrics based on Threshold and Calibration Plots


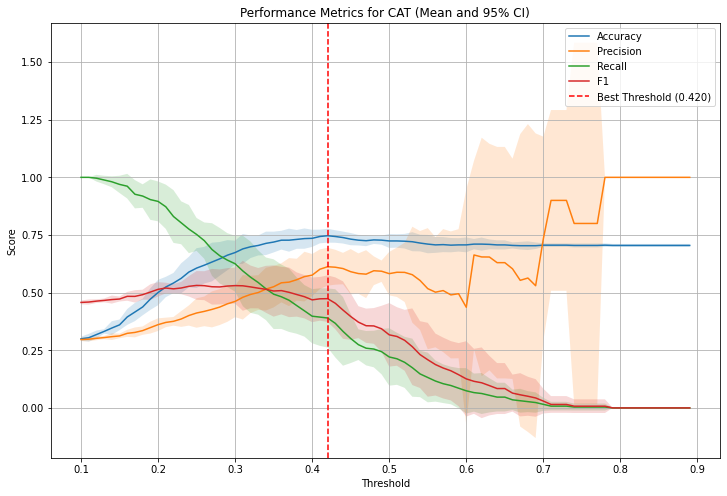

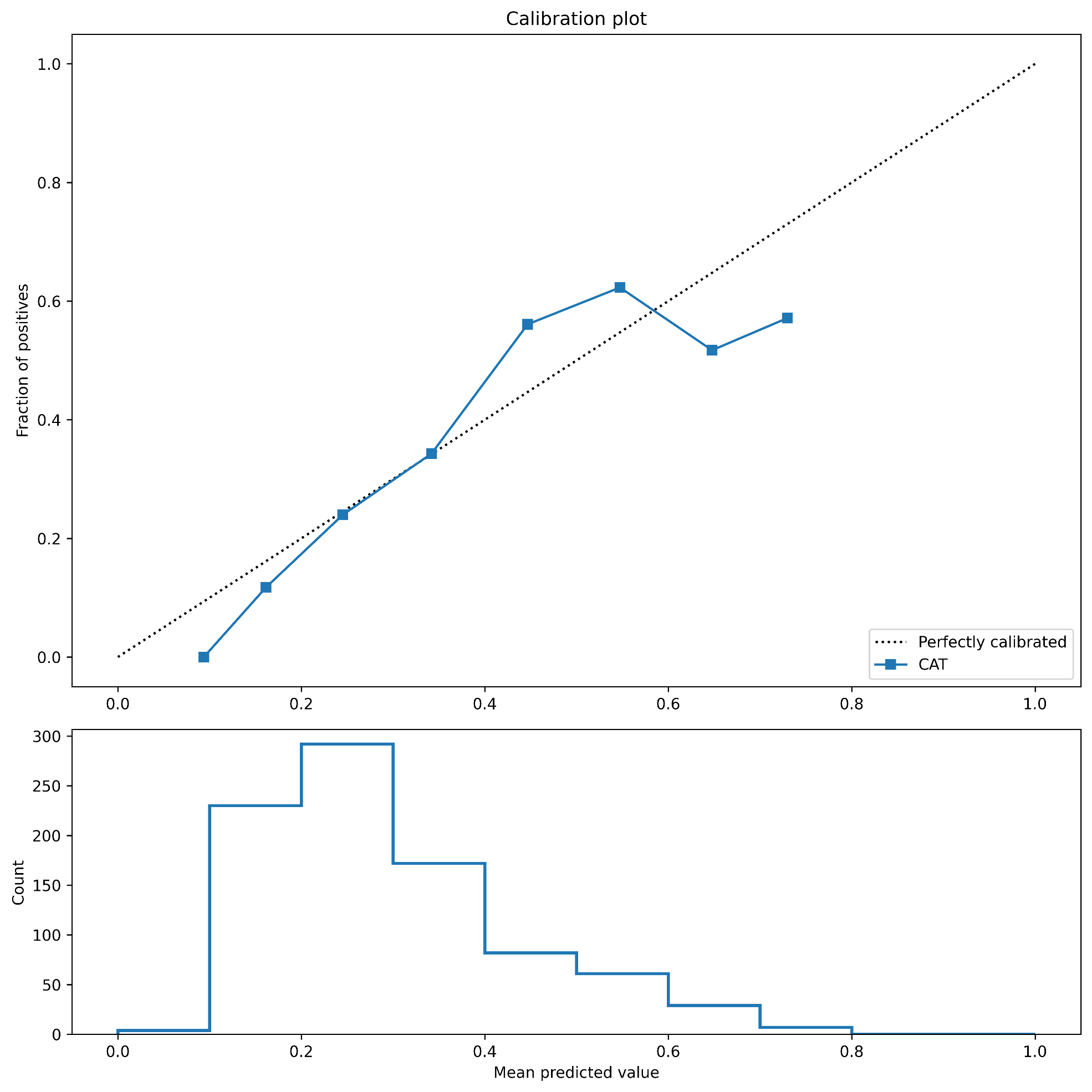


Supplementary Material 9. Performance Comparison of the Prediction of Acute Kidney Injury in Pesticide Intoxication (PKIP) Model Across Different Pesticide Types

| **Pesticide Category** | **Total** | **AKI** (%) | **Death^b^** (%) | **Accuracy**  **(95% CI)** | **Precision**  **(95% CI)** | **Recall**  **(95% CI)** | **F1**  **(95% CI)** | **AUROC**  **(95% CI)** | **AUPRC**  **(95% CI)** |
| --- | --- | --- | --- | --- | --- | --- | --- | --- | --- |
| Total | 877 | 259  (0.3) | 72  (0.08) | 0.747  (0.726-0.768) | 0.612  (0.554-0.671) | 0.390  (0.299-0.481) | 0.474  (0.402-0.545) | 0.720  (0.692-0.747) | 0.513  (0.464-0.563) |
| Other pesticides ^a^ (e.g., pyrethroid) | 321  (0.37) | 70  (0.21) | 20  (0.06) | 0.791  (0.751-0.832) | 0.537  (0.356-0.719) | 0.271  (0.156-0.387) | 0.359  (0.216-0.502) | 0.710  (0.670-0.749) | 0.433  (0.391-0.474) |
| Glufosinate | 216  (0.25) | 76  (0.35) | 21  (0.01) | 0.676  (0.602-0.750) | 0.583  (0.371-0.795) | 0.368  (0.276-0.461) | 0.445  (0.341-0.550) | 0.685  (0.521-0.848) | 0.527  (0.385-0.669) |
| Glyphosate | 223  (0.25) | 61  (0.27) | 15  (0.07) | 0.780  (0.727-0.833) | 0.690  (0.513-0.866) | 0.344  (0.195-0.493) | 0.455  (0.286-0.623) | 0.711  (0.619-0.804) | 0.557  (0.440-0.675) |
| Organo / Carbamate | 117  (0.13) | 52  (0.44) | 16  (0.13) | 0.693  (0.611-0.775) | 0.682  (0.533-0.830) | 0.636  (0.487-0.785) | 0.647  (0.567-0.726) | 0.680  (0.621-0.739) | 0.650  (0.552-0.748) |

Each performance metric was calculated using the mean and 95% confidence interval (CI) from 5-fold cross-validation.

AKI, Acute Kidney Injury, AUROC, Area Under the Receiver Operating Characteristic curve; AUPRC, Area Under the Precision-Recall Curve; CI, confidence interval

^b^ Death refers to all-cause mortality.

^a^ Other pesticides include acetanilide, acetylaniline, alryoxylcarboxide, amide, anilin, arsenic, phenopropionate, benzohydrazide, benzoate, chlorfenapyr, chloroacetamide, chloronicotinyl, diamide, diazine, dinitroaniline, endosulfan, fungicide, insect growth regulator, lambda cyhalothrin, neonicotinoid, niacin, oxadiazole, phenoxy, pyrol, sulfonylurea, sulfoximine, sulfuryl fluoride, tetramic acid, tetrazolium oxide, and unknown pesticides.

Supplementary Material 10. The Prediction of acute Kidney Injury in Pesticide intoxication (PKIP) model Probability distribution

**
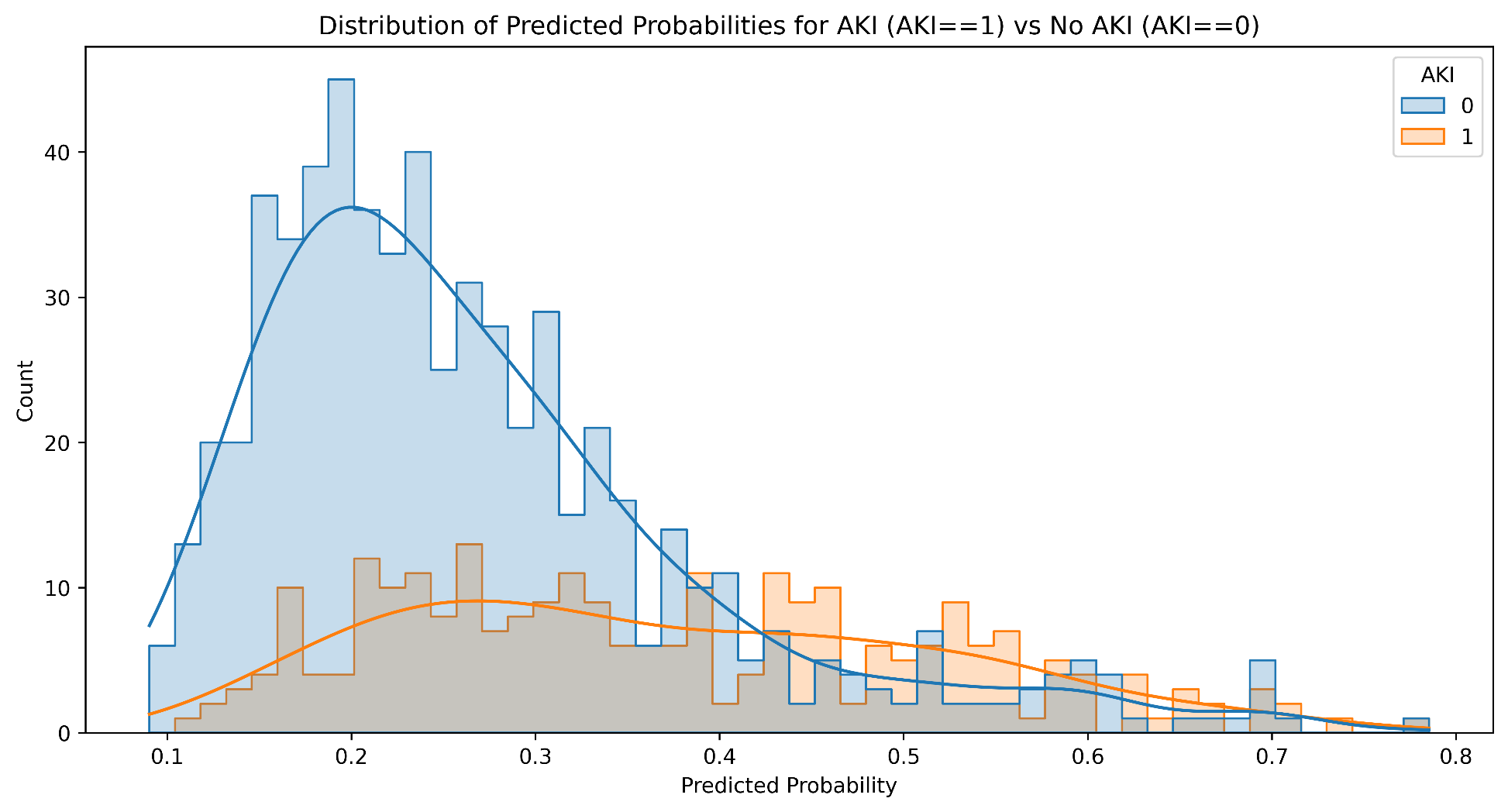
**

Supplementary Material 11. Risk stratification and outcomes using the APACHE II score
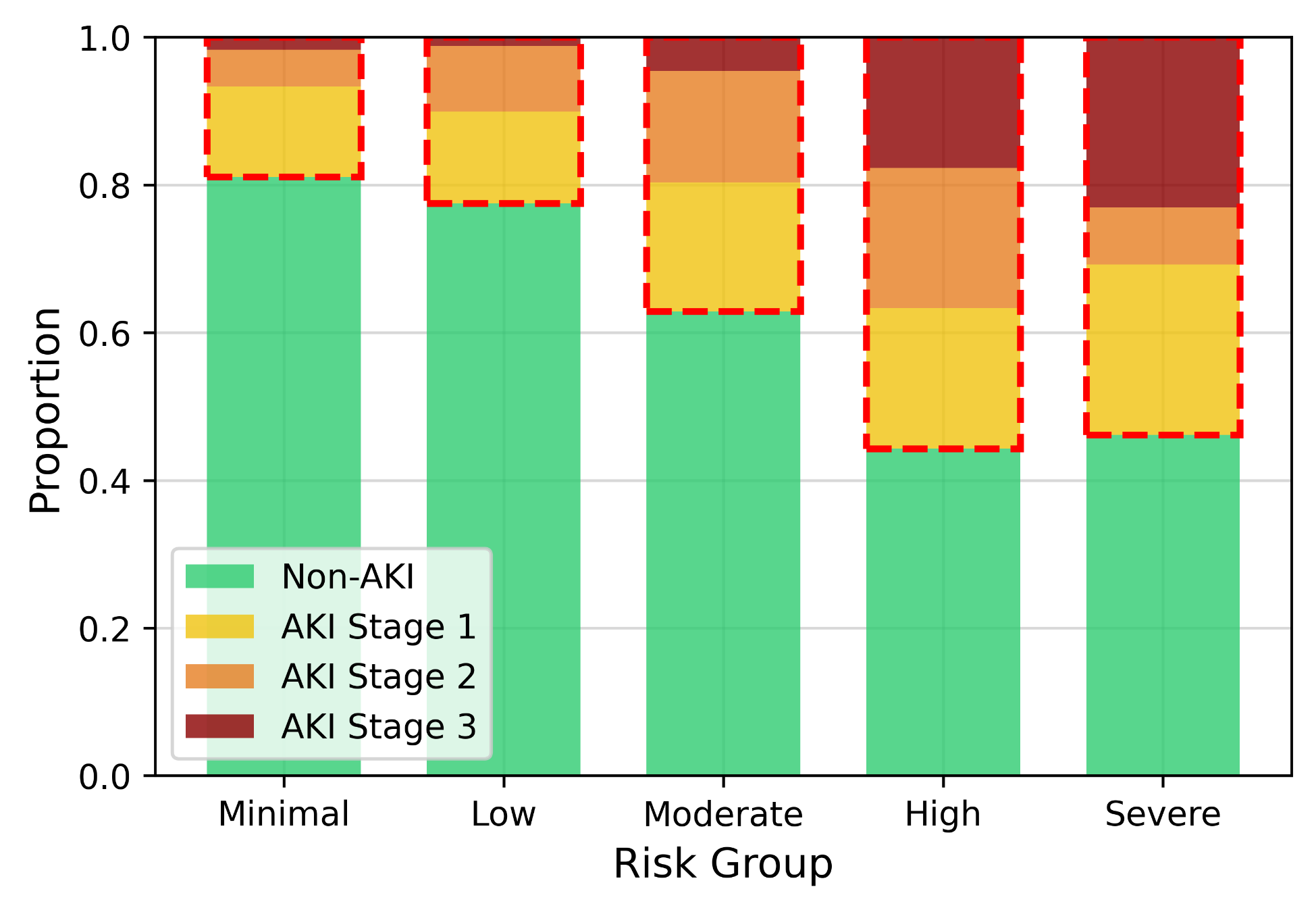


| **Score** | **0 - 4** | **5 - 9** | **10 - 19** | **20 - 29** | **30+** | **Trend test** |
| --- | --- | --- | --- | --- | --- | --- |
| **Risk Group** | **Minimal** | **Low** | **Moderate** | **High** | **Severe** |  |
| **Total** | 180 (0.21) | 338 (0.39) | 264 (0.30) | 79 (0.09) | 13 (0.01) |  |
| **AKI, yes(%)** | 34 (0.19) | 76 (0.22) | 98 (0.37) | 44 (0.56) | 7 (0.54) | 0.091 |
| **stage 1** | 22 | 42 | 46 | 15 | 3 |  |
| **stage 2** | 9 | 30 | 40 | 15 | 1 |  |
| **stage 3** | 3 | 4 | 12 | 14 | 3 |  |
| **Death, yes(%)** | 1 (0.01) | 8 (0.02) | 27 (0.10) | 28 (0.35) | 8 (0.62) | 0.205 |

AKI, Acute Kidney Injury, ^b^ Death refers to all-cause mortality.

^b^ Death refers to all-cause mortality.

* indicates *p*-value < 0.05

** indicates *p*-value < 0.01

*** indicates *p*-value < 0.001

Supplementary Material 12. Risk stratification and survival analysis using the APACHE II score

**
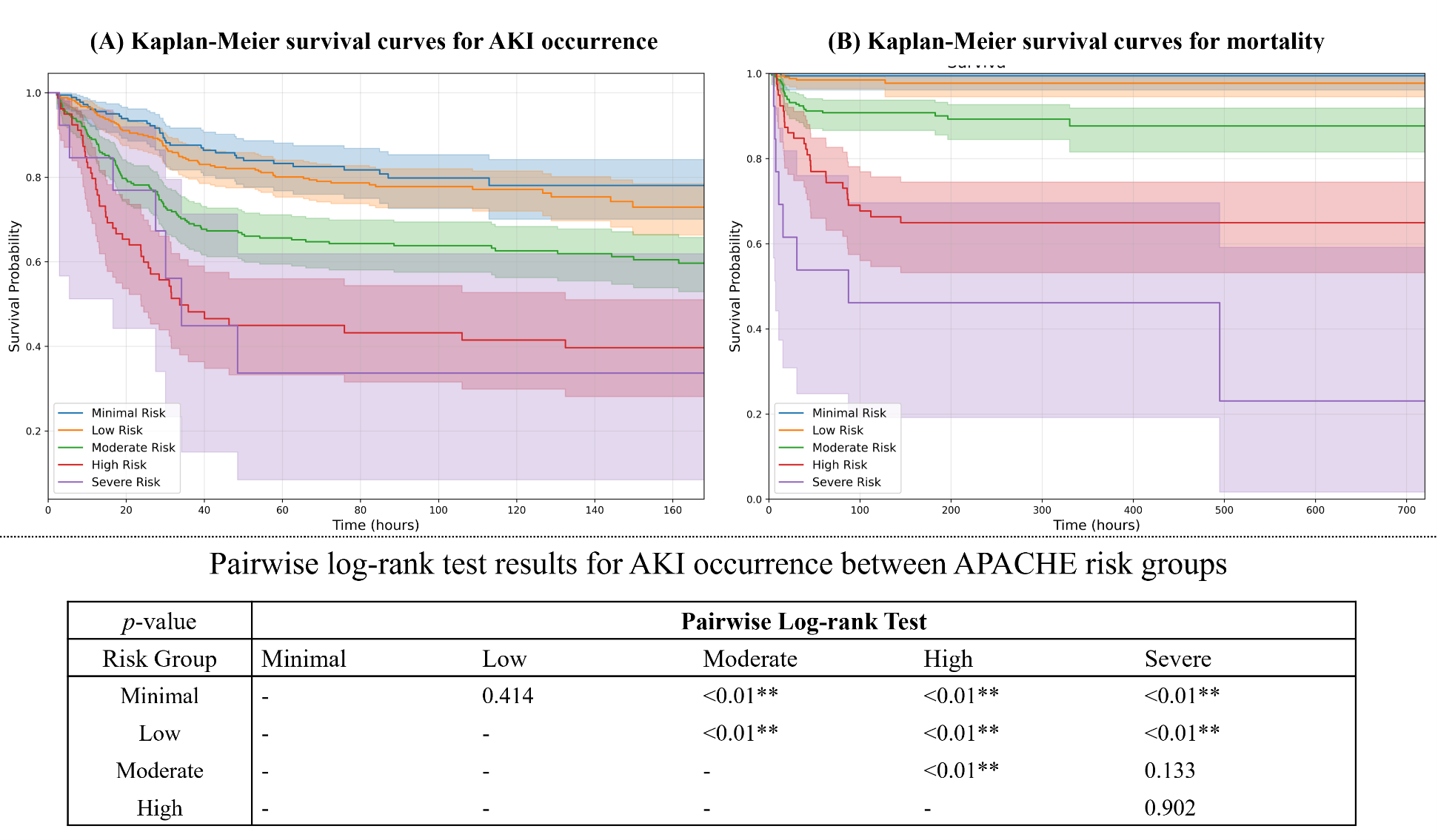
**

Supplementary Material 13. Performance Comparison Between APACHE II model and Prediction of Acute Kidney Injury in Pesticide Intoxication Models for Predicting Acute Kidney Injury

| **Target** | **APACHE II model** | | **PKIP model** | |
| --- | --- | --- | --- | --- |
|  | **AUROC**  **(95% CI)** | **AUPRC**  **(95% CI)** | **AUROC**  **(95% CI)** | **AUPRC**  **(95% CI)** |
| **AKI** | 0.673  (0.603 - 0.744) | 0.459  (0.375 - 0.542) | 0.720  (0.692 - 0.747) | 0.513  (0.464 - 0.562) |
| **Death^a^** | 0.829  (0.762 - 0.896) | 0.331  (0.190 - 0.472) | 0.839  (0.767 - 0.910) | 0.421  (0.246 - 0.595) |

Each performance metric was calculated using the mean and 95% confidence interval (CI) from 5-fold cross-validation. AKI, Acute Kidney Injury, AUROC, Area Under the Receiver Operating Characteristic curve; AUPRC, Area Under the Precision-Recall Curve; CI, confidence interval

^a^ Death refers to all-cause mortality.

Supplementary Material 14. Comparison of Creatinine Variation and Mortality Within 168 Hours After Admission According to AKI Classification

|  | **Non-AKI**  **(*n* = 618)** | **Ambiguous AKI**  **(*n* = 60)** | **Strict AKI**  **(*n* = 199)** | ***P*-value** |
| --- | --- | --- | --- | --- |
| **Creatinine range ^a^** | 0.20 [0.10-0.35] | 0.50 [0.40-0.60] | 0.48 [0.30-0.80] | < 0.001 |
| **Mortality** | 29 (4.69%) | 4 (6.67%) | 39 (19.60%) | < 0.001 |

Baseline serum creatinine was determined dynamically during hospitalization. When any creatinine value decreased by ≥ 0.3 mg/dL compared with the minimum value obtained within 2 hours after admission, those values were either included (Ambiguous AKI) or excluded (Strict AKI) as potential baseline serum creatinine candidates before applying the KDIGO criteria.

^a^ Creatinine range was calculated as the difference between the maximum and minimum serum creatinine levels measured within 168 hours after admission.

Supplementary Material 15. Model Performance According to AKI Definition and Censoring Strategy

| **Case** | | **AKI** | **Non-AKI** | **AUROC** | **AUPRC** |
| --- | --- | --- | --- | --- | --- |
| **Ambiguous AKI** | **Early censored** |  |  |  |  |
| **AKI** | **Included** | 259 | 618 | 0.7171 | 0.5041 |
| **Non-AKI** | **Included** | 199 | 678 | 0.7300 | 0.4372 |
| **Excluded** | **Included** | 199 | 618 | 0.7409 | 0.4725 |
| **AKI** | **Excluded** | 259 | 590 | 0.7313 | 0.5537 |
| **Non-AKI** | **Excluded** | 199 | 613 | 0.7438 | 0.4979 |
| **Excluded** | **Excluded** | 199 | 590 | 0.7550 | 0.5262 |

Ambiguous AKI refers to cases in which serum creatinine values that decreased by ≥0.3 mg/dL compared with the minimum value obtained within 2 hours after admission were included as potential baseline candidates when applying the KDIGO criteria. “Early censored” indicates patients who died within 168 hours after admission without developing AKI. The configuration in which Ambiguous AKI was treated as AKI and Early censored cases were included represents the most conservative evaluation, as reported in the main text.

AKI, Acute Kidney Injury, AUROC, Area Under the Receiver Operating Characteristic curve; AUPRC, Area Under the Precision-Recall Curve.
