# Supplementary material for: Prediction of acute kidney injury in patients with acute pesticide poisoning using the PKIP score
Source: Sci Rep. 2026 Mar 26;16:15086. doi: 10.1038/s41598-026-41334-4 (PMC13172389; doi:10.1038/s41598-026-41334-4)

AKI Prediction Software for Pesticide Poisoning Patients

Pesticide Intoxication

- [ ] Organophosphate, Carbamate or Glufosinate

Patient Information

Age, years: 43

Body Mass Index, kg/m2: 21.9

- [ ] Chronic kidney disease, present

Vital Signs

Body temperature, °C: 36.8

Glasgow Coma Scale: 3

Medical Information (Select if applicable)

- [ ] Hypoxemia, pO2 ≤ 60
- [ ] Urine RBC ≥ 1

Lab Test Results

Bicarbonate, mmol/L: 24

Phosphate, mg/dL: 3.5

White Blood Cell count, $10^3/\mu L$: 7.5

Hemoglobin, g/dL: 14

Anion gap, mmol/L: 12

Alkaline phosphatase, U/L: 80

Predict

The predicted risk group is **Severe** risk group.

AKI Risk: 61%

Mortality Risk with AKI: 30%

The reasons for this assessment are outlined below.

Risk Group Distribution

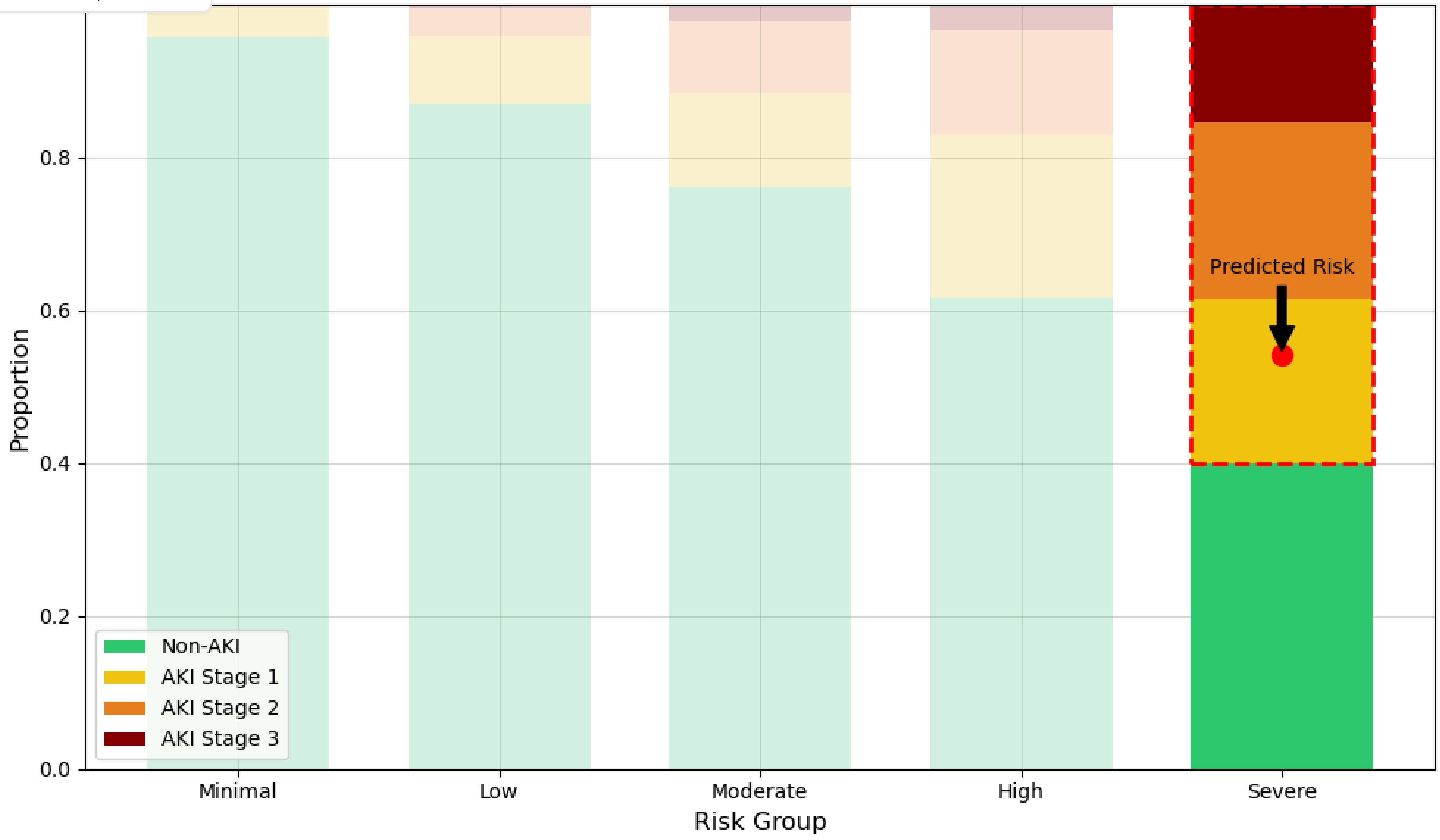

Risk Group Summary

Based on data from the Pesticide Poisoning Center (Cheonan Hospital), there are a total of 143 patients (16.00%) in the Severe risk group out of 877 patients. In the Severe risk group, there are 86 patients with AKI (60.00%) and 45 patients who died (31.00%).

SHAP Explanation

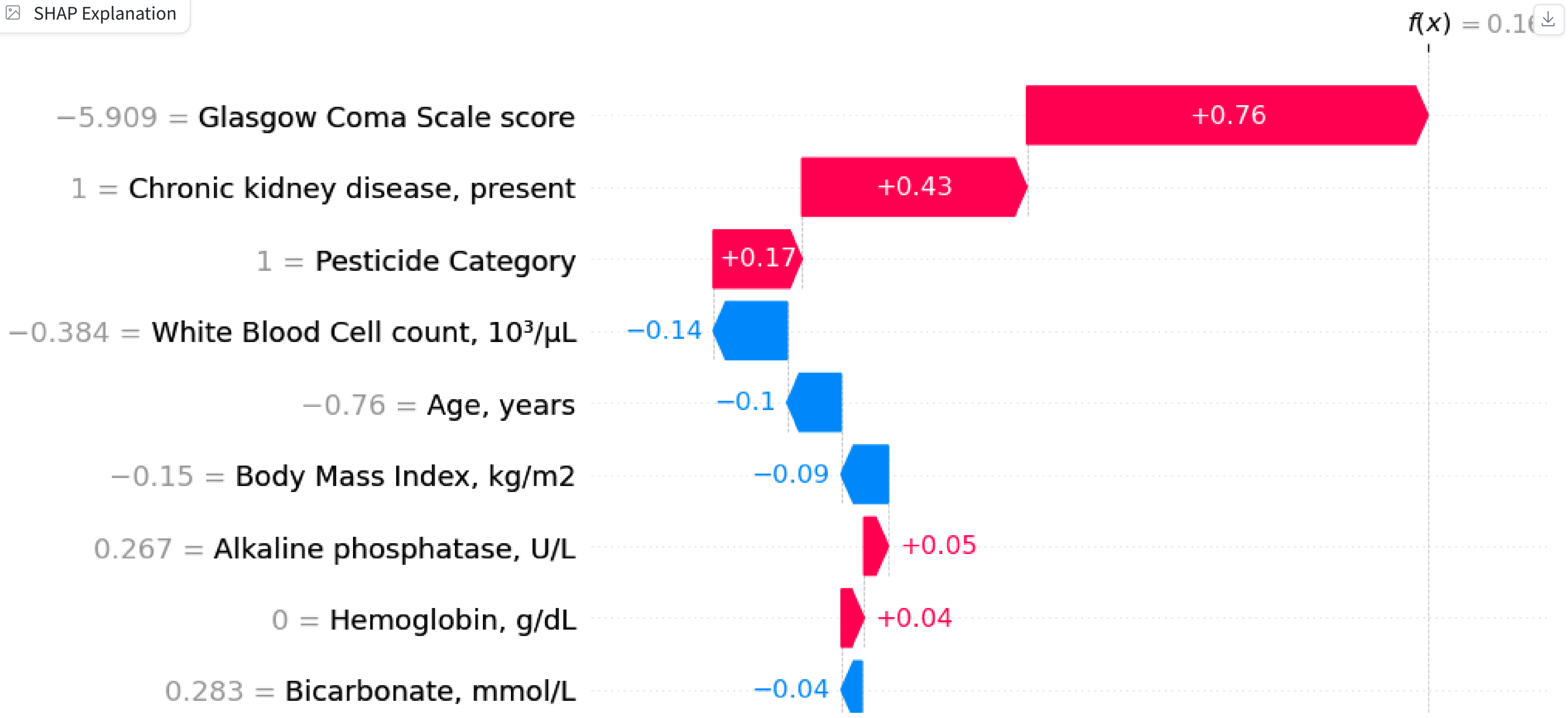

Supplement: Supplementary file 2 — Supplementary Material 2 [file 41598_2026_41334_MOESM2_ESM.pdf]
